# ID2 and HIF-1**α** collaborate to protect quiescent hematopoietic stem cells from activation, differentiation, and exhaustion

**DOI:** 10.1172/JCI152599

**Published:** 2022-07-01

**Authors:** Brad L. Jakubison, Tanmoy Sarkar, Kristbjorn O. Gudmundsson, Shweta Singh, Lei Sun, Holly M. Morris, Kimberly D. Klarmann, Jonathan R. Keller

**Affiliations:** 1 Basic Science Program, Frederick National Laboratory for Cancer Research, Frederick, Maryland, USA.; 2 Mouse Cancer Genetics Program, Center for Cancer Research, National Cancer Institute (NCI) – Frederick, NIH, Frederick, Maryland, USA.

**Keywords:** Hematology, Stem cells, Hematopoietic stem cells

## Abstract

Defining mechanism(s) that maintain tissue stem quiescence is important for improving tissue regeneration, cell therapies, aging, and cancer. We report here that genetic ablation of *Id2* in adult hematopoietic stem cells (HSCs) promotes increased HSC activation and differentiation, which results in HSC exhaustion and bone marrow failure over time. *Id2*^Δ/Δ^ HSCs showed increased cycling, ROS production, mitochondrial activation, ATP production, and DNA damage compared with *Id2^+/+^* HSCs, supporting the conclusion that *Id2*^Δ/Δ^ HSCs are less quiescent. Mechanistically, HIF-1α expression was decreased in *Id2*^Δ/Δ^ HSCs, and stabilization of HIF-1α in *Id2*^Δ/Δ^ HSCs restored HSC quiescence and rescued HSC exhaustion. Inhibitor of DNA binding 2 (ID2) promoted HIF-1α expression by binding to the von Hippel-Lindau (VHL) protein and interfering with proteasomal degradation of HIF-1α. HIF-1α promoted *Id2* expression and enforced a positive feedback loop between ID2 and HIF-1α to maintain HSC quiescence. Thus, sustained ID2 expression could protect HSCs during stress and improve HSC expansion for gene editing and cell therapies.

## Graphical abstract

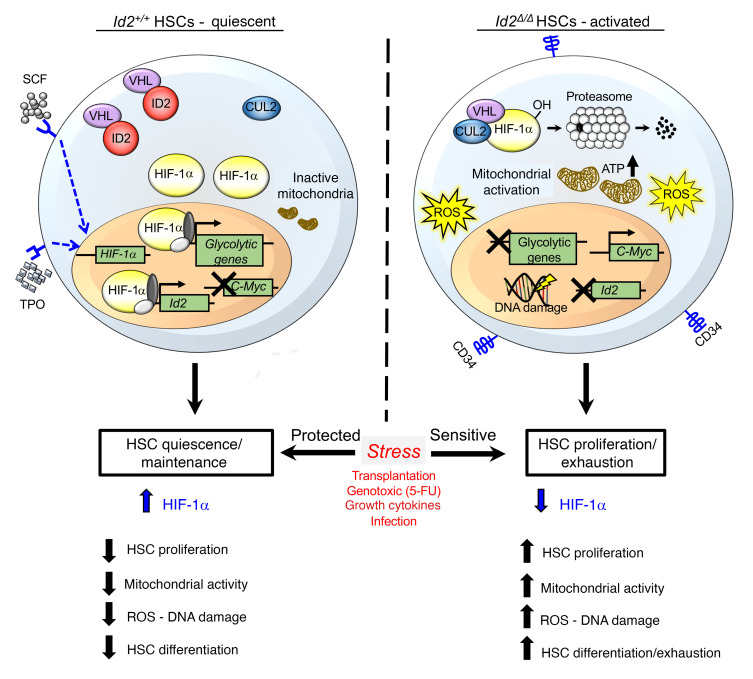

## Introduction

Adult hematopoiesis is sustained by a limited number of hematopoietic stem cells (HSCs) that proliferate to self-renew or differentiate to give rise to multipotent progenitor cells (MPPs), which maintain normal numbers of differentiated blood cells (1, 2). HSCs predominantly reside in a quiescent state, protected from proliferation-induced damage and exhaustion (3, 4). Recent evidence suggests that HSCs have a limited number of self-renewal divisions, suggesting that HSC quiescence, proliferation, and differentiation are tightly regulated processes that prevent HSC exhaustion and hematopoietic failure (5, 6). Deregulation of these processes contributes to clonal expansion, which frequently progresses to hematopoietic malignancies (7). The choice between HSC proliferation, self-renewal, and differentiation is regulated extrinsically by cytokines produced by cells in the hematopoietic microenvironment (HME) and intrinsically by proteins and pathways that regulate gene expression (8, 9). Although there has been significant progress in identifying epigenetic and transcriptional regulators of normal and malignant hematopoiesis, our understanding of the precise molecular mechanism or mechanisms that regulate HSC quiescence and self-renewal is incomplete. Defining these mechanism(s) will lead to better strategies to expand HSCs for cell and gene therapies and to develop targeted therapies to reduce the onset and development of leukemia.

Inhibitor of DNA-binding proteins (ID1–4) are helix-loop-helix transcriptional regulators that modulate cell proliferation and differentiation in numerous tissues including muscle, neuronal, endothelial, and hematopoietic tissue (10–13). ID proteins regulate the growth of embryonic and adult tissue stem cells and are frequently overexpressed in cancer and cancer stem cells (10, 14, 15). The founding member of this family, ID1, is expressed at low levels in HSCs but is induced during stress (16–19). ID1 promotes HSC proliferation and myeloid differentiation, while suppressing lymphoid development through E protein inhibition (17, 18, 20–22). Ablation of *Id1* protects HSCs from proliferative exhaustion during bone marrow transplantation (BMT), inflammatory stress, and aging. In comparison, ID2 is expressed in murine hematopoietic stem and progenitor cells (HSPCs), attenuates B cell development, and promotes myeloid, NK cell, and DC differentiation by inhibiting E protein function (17, 23–28). ID2 is expressed in human HSCs, and enforced expression of ID2 promotes the expansion of primitive CD34^+^CD38^–^cells, which suggests that ID2 may regulate HSC quiescence and proliferation (29). Therefore, we investigated the cell-intrinsic requirement of ID2 in HSCs using mouse models in which Id2 was conditionally deleted and Id2 reporter mice to evaluate HSPC populations that express ID2.

We report here that ID2 is intrinsically required for self-renewal and maintenance of adult HSCs. Loss of *Id2* reduced HSC quiescence and increased HSC activation, which are functions distinct from those of ID1. *Id2*^Δ/Δ^ HSCs exhaust over time, resulting in BM failure and reduced survival. HIF-1α levels were markedly reduced in isolated *Id2*^Δ/Δ^ HSCs. Increasing HIF-1α expression in *Id2*^Δ/Δ^ HSCs restored HSC quiescence and rescued HSC exhaustion in vitro and in vivo. ID2 stabilized HIF-1α expression by binding to von Hippel-Lindau (VHL), which interfered with HIF-1α degradation, providing an alternative mechanism to low oxygen levels to stabilize and increase HIF-1α expression. Furthermore, HIF-1α directly regulated *Id2* transcription through identified HIF-1 response element (HRE) CACA sites in the *Id2* promoter. Thus, HIF-1α and ID2 enforce each other’s expression, creating a positive feedback loop to maintain quiescent HSCs and prevent HSC exhaustion.

## Results

### ID2 maintains HSCs during steady-state hematopoiesis.

**Figure 1:**
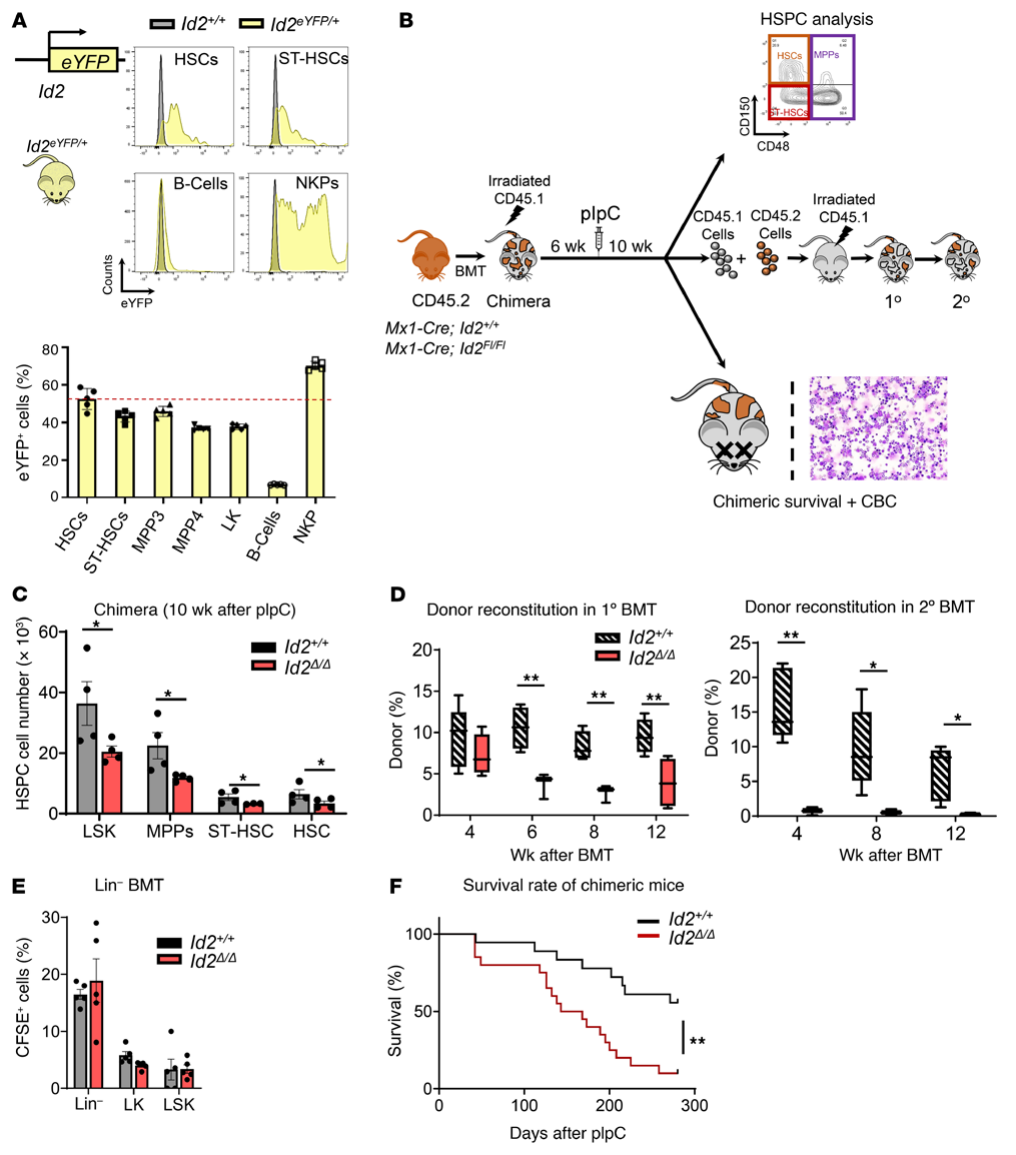
ID2 is intrinsically required to maintain HSCs in vivo. (**A**) Flow cytometric histograms of ID2/eYFP (upper panels) and the percentage of ID2/eYFP expression in HSPCs, B cells, and NK progenitors (lower panel). (**B**) Summary of the analysis of *Mx1-Cre* *Id2^+/+^* (*Id2^+/+^*) and *Mx1-Cre* *Id2^fl/fl^* (*Id2*^Δ/Δ^) chimeric mice. (**C**) Total number of HSPCs in BMCs from chimeric mice 10 weeks after administration of pIpC. (**D**) Donor reconstitution of primary competitive and secondary BMT recipient mice. (**E**) HSPC analysis of CFSE-labeled *Id2^+/+^* and *Id2*^Δ/Δ^ Lin^–^ BMCs (2 × 10^6^) transplanted into CD45.1 recipient mice 20 hours after BMT. (**F**) Survival of *Id2^+/+^* and *Id2*^Δ/Δ^ chimeric mice after pIpC treatment. In **C** and **E**, data are presented as the mean ± SEM, and comparisons between mean values of 2 groups were evaluated using an unpaired, 1-tailed Student’s *t* test. In **D**, the center line indicates the median and the box represents the 25th and 75th percentiles, and statistical significance was determined by unpaired, 1-tailed Student’s *t* tests. Kaplan-Meier survival studies were analyzed using Wilcoxon’s signed-rank. *P ≤ 0.05 and **P ≤ 0.01.

*Id2* is expressed in HSPCs and regulates lineage commitment and the differentiation of primitive and more differentiated progenitors (17, 29); however, the precise function of *Id2* in regulating HSC development has not, to our knowledge, been examined. Using an *Id2* reporter mouse model (*Id2^eYFP/+^*), we confirmed that ID2 is expressed in HSCs, short-term HSCs (ST-HSCs), and in MPP populations and show that roughly half of HSCs express ID2^eYFP^ (Figure 1A and Supplemental Figure 1, A–D; supplemental material available online with this article; https://doi.org/10.1172/JCI152599DS1). We observed no difference in the number of HSCs or MPPs or in the development of myeloid, B, or erythroid cells in *Id2^eYFP/+^* and *Id2^+/+^* mice (data not shown). As expected, B cells showed little or no ID2^eYFP^ expression, whereas NK progenitors expressed high levels of ID2 (Figure 1A and refs. 30–33).

To examine the intrinsic requirement for *Id2* in HSCs we developed an *Id2*-conditional (*Id2^fl/fl^*) mouse model (Supplemental Figure 1, E–H) and bred these mice with *Mx1-Cre*–transgenic mice. BM cells (BMCs) from *Mx1-Cre* *Id2^fl/fl^* and control mice (*Mx1-Cre* *Id2^+/+^*) were transplanted into γ-irradiated (γ-IR) mice (chimeric mice) to eliminate any contribution that loss of *Id2* function may have in the HME. Mice were treated with polyinosinic:polycytidylic acid (pIpC) 6 weeks after BMT to ablate *Id2* expression (*Id2*^Δ/Δ^) in hematopoietic lineage cells and then examined the mice for hematopoietic development under steady-state conditions (Figure 1B). Ten weeks after pIpC treatment, we observed a significant decrease in the number of immunophenotypic HSCs (Lin^–^Sca1^+^c-Kit^+^ [LSK] FLT3^–^CD150^+^CD48^–^), ST-HSCs (LSK FLT3^–^CD150^–^CD48^–^), and MPPs (LSK FLT3^–^CD34^+^) in *Id2*^Δ/Δ^ chimeric mice compared with control mice (Figure 1C). Donor reconstitution was significantly reduced in primary and secondary competitively transplanted mice that received *Id2*^Δ/Δ^ BMCs compared with controls, demonstrating functional loss of HSCs in *Id2*^Δ/Δ^ chimeric mice (Figure 1D). Immunophenotype analysis of primary competitively transplanted mice showed a significant decrease in immunophenotypic HSCs and downstream progenitor cells compared with controls (Supplemental Figure 1I). Reduced HSC function was not due to a defect in the ability of *Id2*^Δ/Δ^ HSPCs to home to BM, since no difference in the number of CFSE-labeled *Id2*^Δ/Δ^ and *Id2^+/+^* Lin^–^ BMCs was observed in the BM of transplanted mice (Figure 1E). Finally, *Id2*^Δ/Δ^ chimeric mice had reduced survival due to anemia, leukocytosis, and BM failure compared with *Id2^+/+^* chimeric mice (60% survival; Figure 1F and Table 1). Taken together, these results demonstrate that ID2 is intrinsically required to maintain HSCs during steady-state hematopoiesis.

### ID2 promotes HSC quiescence.

**Figure 2:**
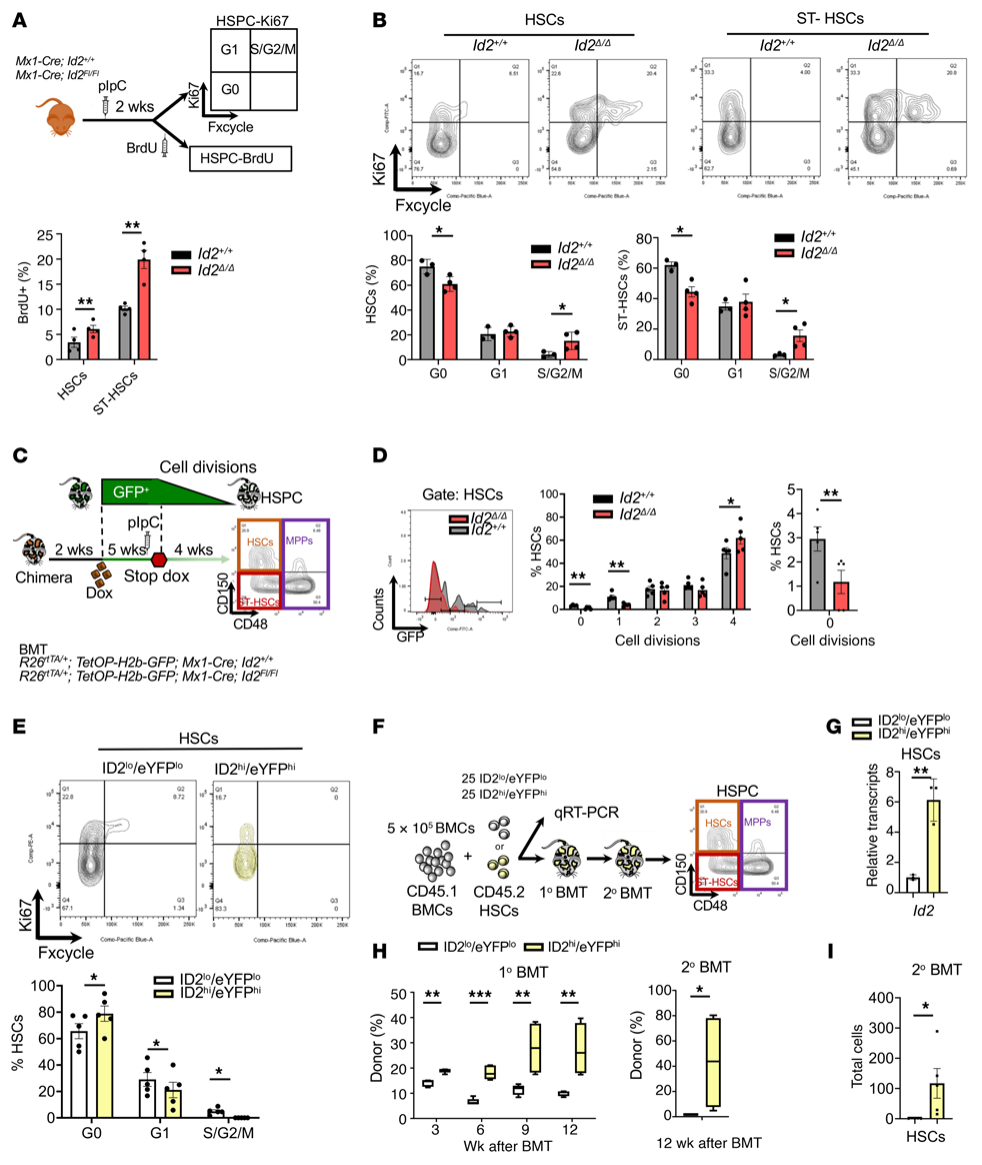
Cell proliferation is increased in *Id2^Δ/Δ^* HSCs in vivo. (**A**) Summary of the procedure to evaluate the proliferation of *Id2*^Δ/Δ^ and *Id2^+/+^* HSCs and ST-HSCs in vivo, 2 weeks after ablation of *Id2*. (**B**) Flow cytometry contour maps of Ki-67 expression in *Id2^+/+^* and *Id2*^Δ/Δ^ HSCs and ST-HSCs and quantification by cell cycle. (**C**) Summary of the procedure to measure HSC divisions in *R26^rtTA/+^* *TetOP-H2B-GFP* *Mx1-Cre* *Id2^fl/fl^* and *R26 ^rtTA/+^* *TetOP-H2B-GFP* *Mx1-Cre* *Id2^+/+^* mice. Mice were given Dox for 6 weeks, starting 2 weeks after BMT, followed by a 4-week chase; pIpC was administered 1 week before the chase. (**D**) GFP retention in HSCs after a 4-week chase. (**E**) Flow cytometry contour maps of Ki-67 expression in ID2^hi^eYFP^hi^ and ID2^lo^eYFP^lo^ HSCs and cell-cycle quantification. (**F**) Procedure to measure the serial competitive repopulation potential of ID2^hi^eYFP^hi^ or ID2^lo^eYFP^lo^ HSCs. (**G**) RT-qPCR analysis of *Id2* expression in ID2^hi^eYFP^hi^ and ID2^lo^eYFP^lo^ HSCs. (**H**) Competitive repopulation of ID2^hi^eYFP^hi^ and ID2^lo^eYFP^lo^ HSCs in primary and secondary BMT recipient mice. (**I**) Total HSCs in secondary BMT recipient mice. In **B**, **D**, **E**, **G**, and **I**, data are presented as the mean ± SEM. In **H**, the center line indicates the median, and the box represents the 25th and 75th percentiles. Comparisons between mean values of 2 groups were evaluated using an unpaired, 1-tailed Student’s *t* test. *P ≤ 0.05, **P ≤ 0.01, and ***P ≤ 0.001.

To understand why *Id2*^Δ/Δ^ HSCs (LSK FLT3^–^CD150^+^CD48^–^) exhaust in vivo, we examined the proliferation and cycling of *Id2*^Δ/Δ^ HSCs in *Mx1-Cre* *Id2^fl/fl^* mice 2 weeks after *Id2* ablation. *Id2*^Δ/Δ^ HSCs showed increased levels of BrdU incorporation after *Id2* ablation compared with *Id2^+/+^* HSCs (Figure 2A). In addition, Ki-67 staining revealed that loss of *Id2* in HSCs caused a significant reduction in cells in the G_0_ phase, with a concomitant increase in cells in G_1_ and S/G_2_/M in *Id2*^Δ/Δ^ (Figure 2B) and chimeric mice (Supplemental Figure 2, A and B), confirming that intrinsic loss of *Id2* promotes HSC proliferation in vivo. A similar reduction in the number ST-HSCs (LSK FLT3^–^CD150^–^CD48^–^) in G_0_ was observed in *Id2*^Δ/Δ^ chimeric mice (Figure 2B). To determine whether the loss of HSCs in vivo was also due to increased apoptosis, we measured annexin V levels, which remained unchanged between *Id2*^Δ/Δ^ and *Id2^+/+^* HSCs (Supplemental Figure 2B). To track HSC division in vivo, we transplanted BMCs from *Mx1-Cre* *Id2^+/+^* *Rosa26^rTA/+^* *TetOP-H2b-GFP* and *Mx1-Cre* *Id2^fl/fl^* *Rosa26^rTA/+^ TetOP-H2b-GFP*–transgenic mice into γ-IR recipient mice (Figure 2C). Chimeric mice were given doxycycline (Dox) chow for 5 weeks to induce GFP expression in all hematopoietic cells, after which mice were treated with pIpC to ablate *Id2* expression and removed from Dox treatment to track HSC division. Immunophenotype analysis of HSCs after a 4-week or 8-week chase period showed a significant increase in the frequency of *Id2*^Δ/Δ^ HSCs that had divided 4 or more times (loss of GFP labeling) compared with *Id2^+/+^* HSCs and a decrease in the frequency of *Id2*^Δ/Δ^ HSCs that had not divided or had undergone a single division compared with *Id2^+/+^* HSCs (GFP labeling retained), demonstrating that HSCs that lacked *Id2* had increased cell division in vivo (Figure 2D and Supplemental Figure 2C). In comparison, HSCs that expressed high levels of ID2 (ID2^hi^eYFP^hi^) in *Id2^eYFP/+^* reporter mice had increased quiescence (G_0_) and decreased cycling (G_1_/S/G_2_/M) compared with HSCs with lower ID2 levels (ID2^lo^eYFP^lo^) (Figure 2E). Collectively these data indicate that ID2 expression is associated with HSC quiescence and that loss of *Id2* in HSCs promotes cycling and reduces quiescence.

Recent experiments tracking the divisional history of HSCs (5) suggest a hierarchical relationship among relatively pure populations of HSCs that is defined by their proliferative history and function. Since the ID2^hi^eYFP^hi^ HSCs showed increased quiescence, we asked if there might be a functional difference between ID2^hi^eYFP^hi^ HSCs and ID2^lo^eYFP^lo^ HSCs that would show distinct levels of ID2/eYFP in serial competitive BMT assays (Figure 2, F and G). We found that 25 FACS-sorted ID2^eYFPhi^ HSCs had significantly increased repopulation potential compared with 25 ID2^eYFPlo^ HSCs in primary recipient mice and that the ID2^hi^eYFP^hi^ HSCs had increased secondary and tertiary repopulation potential, indicating that these cells were enriched for self-renewing HSCs and may represent a functionally distinct HSC population (Figure 2H). In agreement with these data, ID2^hi^eYFP^hi^ HSCs were retained in the secondary BMT, while ID2^lo^eYFP^lo^ HSCs were largely absent (Figure 2I).

### Id2^Δ/Δ^ HSCs are sensitive to proliferative stress.

**Figure 3:**
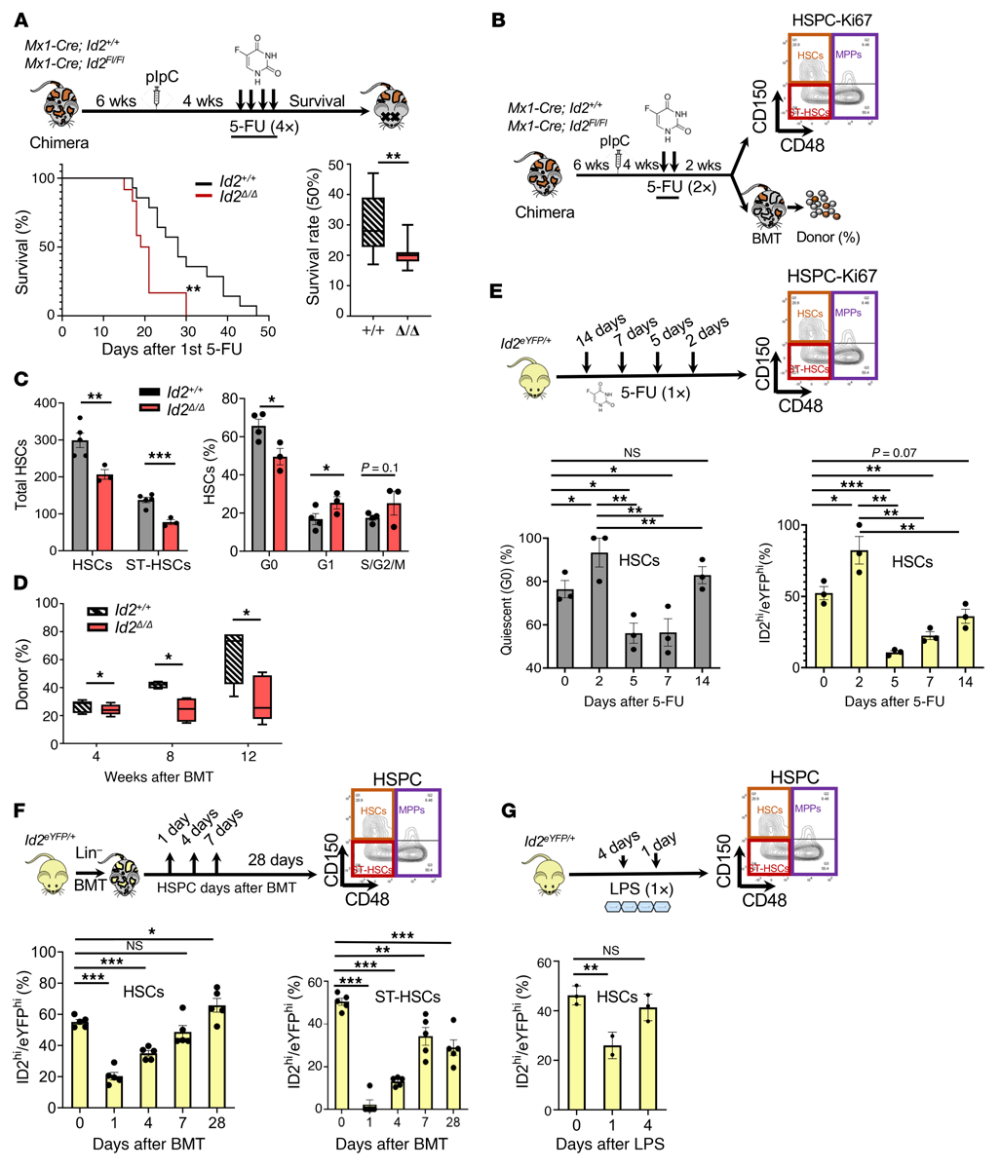
*Id2^Δ/Δ^* HSCs display increased susceptibility to genotoxic stress and reduced *Id2* expression in HSCs after BMT and proliferative stress. (**A**) Summary of the procedure to measure the sensitivity of chimeric *Id2^+/+^* and *Id2*^Δ/Δ^ mice to genotoxic stress following 4 weekly injections of 5-FU (135 mg/kg) and survival of the treated mice. (**B**) Procedure to determine the effect of genotoxic stress on the number, function, and cycling of HSPCs in chimeric *Id2^+/+^* and *Id2*^Δ/Δ^ mice 2 weeks after 2 sublethal injections of 5-FU (150 mg/kg). (**C**) Total cells and Ki-67 cell-cycle analysis of HSCs after administration of 5-FU (2×). (**D**) Analysis of donor reconstitution and HSC function of transplanted BMCs 2 weeks after 2 sublethal injections of 5-FU (150 mg/kg). (**E**) Procedure to examine HSCs in *Id2^eYFP/+^* reporter mice 2, 5, 7, and 14 days after 5-FU treatment; frequency of quiescent (G_0_) HSCs by Ki-67 expression; and ID2^hi^eYFP^hi^ expression in HSCs. (**F**) Procedure to evaluate ID2/eYFP expression in HSCs after transplantation of *Id2^eYFP/+^* BMCs (upper panel) and expression of ID2/eYFP in HSCs and ST-HSCs following BMT. (**G**) Procedure to measure ID2/eYFP expression in HSCs from LPS-treated *Id2^eYFP/+^* mice and expression of ID2/eYFP in HSCs. In **C**, **E**, **F**, and **G**, data are presented as the mean ± SEM. Comparisons between mean values of 2 groups were evaluated using an unpaired, 1-tailed Student’s *t* test. In **A** and **D**, the center line indicates the median, and the box represents the 25th and 75th percentiles. For **E**, 1-way ANOVA with Tukey’s correction was performed, and for **F** and **G**, ANOVA with Dunnett’s correction was used to compare multiple means. Kaplan-Meier survival studies were analyzed using Wicoxon’s signed-rank test.*P ≤ 0.05, **P ≤ 0.01, and ***P ≤ 0.001.

Since *Id2*^Δ/Δ^ HSCs show increased proliferation in vivo, we reasoned that they would be sensitive to the genotoxic stress of 5-fluorouracil (5-FU), which eliminates cycling HSPCs in vivo. Therefore, we treated *Id2*^Δ/Δ^ and *Id2^+/+^* chimeric mice with weekly sublethal doses of 5-FU and found that *Id2*^Δ/Δ^ mice did not survive beyond 30 days after the initial 5-FU treatment compared with *Id2^+/+^* chimeric mice over the same period. This demonstrated that *Id2*^Δ/Δ^ HSCs had increased sensitivity to the cytotoxic effects of 5-FU in vivo (Figure 3A). Immunophenotype analysis of *Id2*^Δ/Δ^ chimeric mice revealed a significant decrease in HSCs and ST-HSCs compared with *Id2^+/+^* mice after 5-FU administration (Figure 3B, summary and Figure 3C, left panel). Furthermore, BMCs from 5-FU–treated *Id2*^Δ/Δ^ mice showed a decreased repopulation ability 12 weeks after competitive BMT, demonstrating an increased loss of functional HSCs in 5-FU–treated *Id2*^Δ/Δ^ chimeric mice compared with controls and confirming that *Id2*^Δ/Δ^ HSCs were more sensitive to the cytotoxic effects of 5-FU (Figure 3D). In addition, *Id2*^Δ/Δ^ HSCs showed an increase in the frequency of HSCs in G_1_ and a decrease in the frequency of HSCs in G_0_ after a second dose of 5-FU compared with frequencies in *Id2^+/+^* HSCs, indicating that *Id2*^Δ/Δ^ HSCs had an increased proliferative response to genotoxic stress and, therefore, were more susceptible to HSC exhaustion following genotoxic stress (Figure 3C, right panel). Since ID2^hi^eYFP^hi^ HSCs were quiescent, we speculated that ID2^eYFPhi^ HSCs would be resistant to the cytotoxic effects of 5-FU. To test this hypothesis, we examined HSCs in *Id2^eYFP/+^* reporter mice 2 days after a sublethal dose of 5-FU and found that the surviving HSCs were largely quiescent (>95%) by Ki-67 staining (Figure 3E and Supplemental Figures 3, A and B) and were enriched for ID2/eYFP-expressing HSCs (80%–90%), suggesting that HSCs with high levels of ID2 were functionally quiescent. We cannot rule out the possibility that ID2/eYFP was upregulated in HSCs that survived 2 days after 5-FU treatment. Five days after 5-FU treatment, when HSCs began to proliferate to repopulate the host, the frequency of quiescent ID2^hi^eYFP^hi^ HSCs was reduced, indicating that ID2 levels decreased as HSCs were recruited into cell cycling in vivo (Figure 3E). The frequency of quiescent ID2/eYFP HSCs increased over the next 2 weeks as the BM recovered and returned to steady state. Thus, we found that ID2/eYFP was expressed in quiescent HSCs and was downregulated when the HSCs were induced to proliferate, after which the HSCs returned to quiescence and upregulated ID2/eYFP expression.

To confirm that ID2/eYFP expression was reduced in HSCs that were induced to proliferate, we transplanted *Id2^YFP/+^* BMCs into γ-IR recipient mice and tracked ID2/eYFP expression in donor HSCs over a 28-day period (Figure 3F). We found that ID2/eYFP expression was significantly reduced in HSCs 1 day after BMT, when HSCs were induced to proliferate, after which ID2/eYFP expression in HSCs slowly recovered to basal levels over the 28-day period, suggesting that HSCs silenced ID2 expression after BMT to repopulate and reconstitute the BM (Figure 3F). Similarly, when mice are administered LPS, which mimics bacterial infection signaling and induces HSC cycling, we found that ID2/eYFP levels were reduced as the HSCs were induced to proliferate and then recovered over a 4-day period (Figure 3G). To confirm that *Id2*^Δ/Δ^ HSCs show increased cycling after BMT, *Mx1-Cre* *Id2^+/+^* *Rosa26^rTA/+^* *TetOP-H2b-GFP* and *Mx1-Cre* *Id2^fl/fl^* *Rosa26^rTA/+^* *TetOP-H2b-GFP* mice were given Dox chow for 6 weeks to induce labeling of BMCs. We treated mice with pIpC 2 weeks before harvesting BMCs for BMT. Mice that were transplanted with *Id2*^Δ/Δ^ BMCs had few HSCs that remained undivided 4 weeks after BMT compared with *Id2^+/+^* mouse HSCs, demonstrating an increase in *Id2*^Δ/Δ^ HSCs proliferation after BMT (Supplemental Figures 2, E–H).

### Id2^Δ/Δ^ HSCs show increased differentiation, reduced quiescence, and increased exhaustion in vitro.

**Figure 4:**
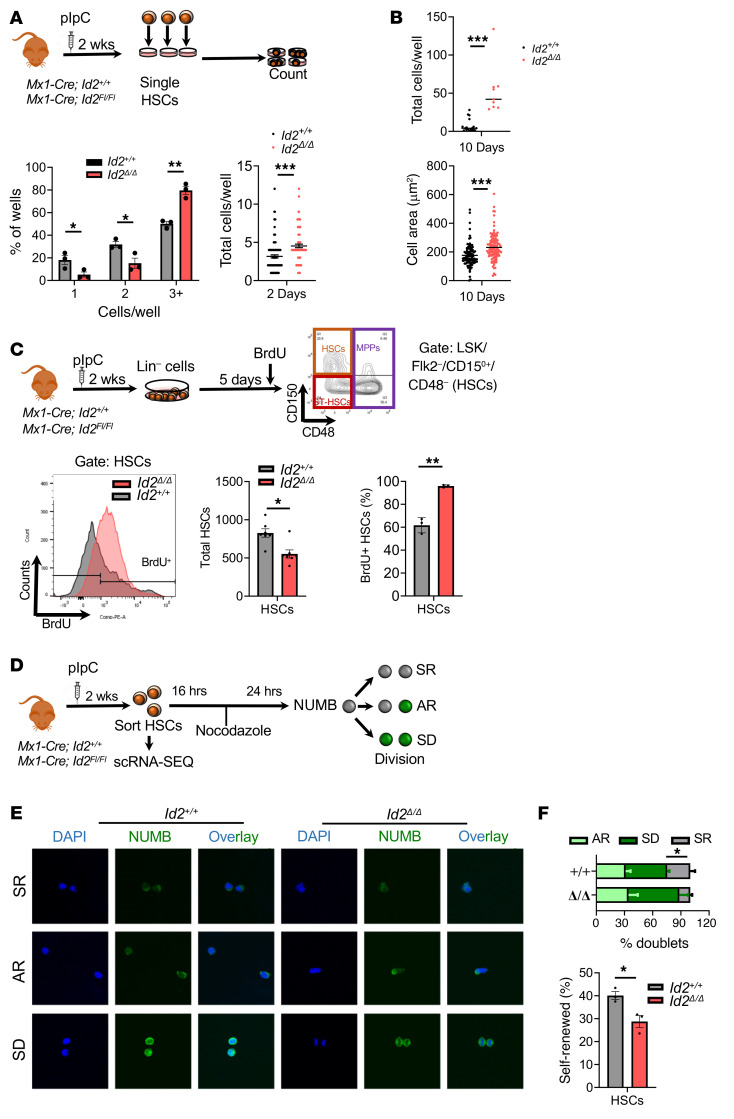
Loss of *Id2* increases HSC proliferation and differentiation. (**A**) Summary of single-cell proliferation assays of FACS-sorted *Id2^+/+^* and *Id2*^Δ/Δ^ HSCs after 48 hours. (**B**) Total cells per well (upper panel) and cell area (lower panel) at 10 days. (**C**) Summary of the method used to analyze BrdU incorporation into *Id2^+/+^*and *Id2*^Δ/Δ^ HSPCs from 5-day Lin^–^ cell expansion cultures. Histogram shows BrdU incorporation into HSCs, total HSCs in culture, and the percentage of BrdU^+^ HSCs. (**D**) Procedure to determine HSC self-renewal by NUMB staining. (**E**) Immunofluorescence analysis of NUMB-stained *Id2^+/+^* and *Id2*^Δ/Δ^ paired daughter HSCs. Original magnification, 40×. (**F**) Quantification of HSC doublets (minimum of 22 daughter cell pairs were counted per genotype in 3 separate experiments) following the NUMB assay and the percentage of self-renewed HSCs after a single division. In **A**, **C**, and **F**, data are presented as the mean ± SEM. In **A** and **B**, column scatter plots show the mean. Comparisons between mean values of 2 groups were evaluated using an unpaired, 1-tailed Student’s *t* test.*P ≤ 0.05, **P ≤ 0.01, and ***P ≤ 0.001. SR, self-renewal; AR, asymmetric renewal; SD, symmetric differentiation.

Since *Id2*^Δ/Δ^ HSCs showed enhanced cycling and reduced numbers in vivo, we speculated that the loss of *Id2*^Δ/Δ^ HSCs may have been a consequence of increased differentiation. Therefore, we evaluated the growth and differentiation of *Id2*^Δ/Δ^ HSCs in vitro. First, we measured the division kinetics of FACS-sorted *Id2^+/+^* and *Id2*^Δ/Δ^ HSCs in response to growth factors in single-cell assays (Figure 4A, summary). We found that the majority (80%) of *Id2*^Δ/Δ^ HSCs had divided more than twice within 48 hours, while significantly fewer (50%) *Id2^+/+^* HSCs had divided more than twice over the same period (Figure 4A, bottom left). *Id2*^Δ/Δ^ HSCs produced more cells per well on average than did *Id2^+/+^* HSCs (Figure 4A, bottom right). Furthermore, after 10 days, we observed that *Id2*^Δ/Δ^ HSCs had produced over 7-fold more cells than did *Id2^+/+^* HSCs (Figure 4B). Thus, *Id2*^Δ/Δ^ HSCs had decreased quiescence and increased proliferation in response to cytokines in vitro.

Since the *Id2*^Δ/Δ^ HSCs showed increased divisional kinetics, we speculated that *Id2*^Δ/Δ^ HSCs might be sensitive to exhaustion. Therefore, we measured HSC exhaustion in expansion assays using Lin^–^ cells and HSPC immunophenotype analysis. Although the total number of HSCs declined in these cultures over 5 days, *Id2*^Δ/Δ^ HSCs showed increased exhaustion compared with *Id2^+/+^* HSCs (Figure 4C). Furthermore, *Id2*^Δ/Δ^ HSCs and ST-HSCs showed increases in both BrdU incorporation (Figure 4C) and Ki-67 staining (Supplemental Figure 4A) compared with control HSCs after 5 days in HSC expansion cultures. In agreement with these data, we observed a 3-fold reduction in human CD34^+^CD38^–^ cells in 3 cord blood samples following knockdown of *ID2* when compared with control lentivirus–infected samples 4 days after infection (Supplemental Figure 4B). Overall, the data suggest that increased proliferation led to increased differentiation and exhaustion of *Id2-*deficient HSCs in both murine and human systems.

To determine whether *Id2*^Δ/Δ^ HSCs show increased differentiation in HSC expansion cultures, we measured the expression of the negative regulator of NOTCH signaling (34) NUMB, which is increased in differentiating HSCs. *Id2*^Δ/Δ^ and *Id2^+/+^* HSCs were sorted into chamber slides and cultured for 16 hours to promote cell division, after which cell division was blocked with nocodazole, and cells were stained for NUMB expression (Figure 4D). We observed a significant reduction in symmetrical self-renewal divisions of *Id2*^Δ/Δ^ HSCs and a concomitant increase in symmetrical differentiation (Figure 4, E and F). Collectively, these data suggest that *Id2*^Δ/Δ^ HSCs underwent increased differentiation in response to cytokines in vitro.

### Id2^Δ/Δ^ HSCs show an activated molecular signature and reduced HIF-1α expression.

**Figure 5:**
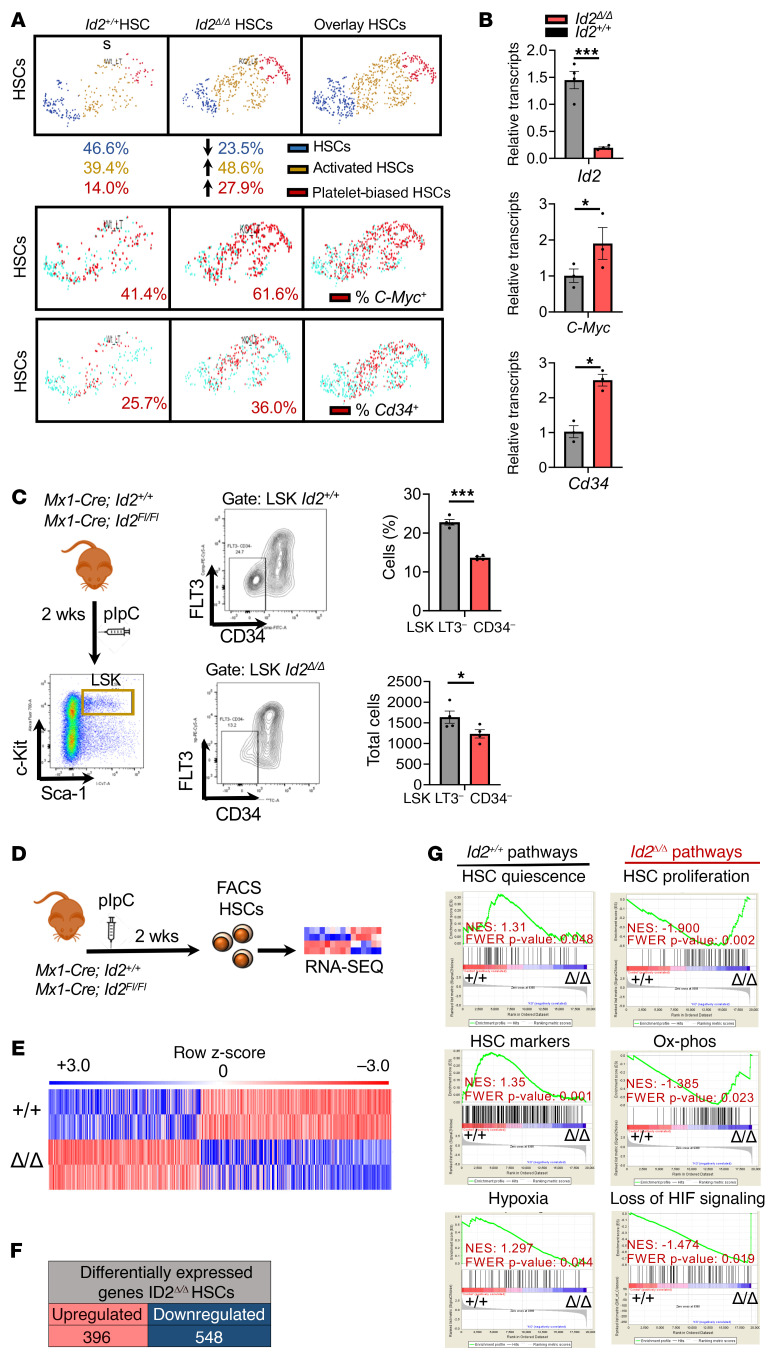
The *Id2^Δ/Δ^* HSC transcriptome shows reduced quiescence, increased oxidative phosphorylation, and loss of HIF signaling. (**A**) scRNA-Seq clustering comparison of HSCs, activated HSCs, and platelet-biased HSCs (top panel), expression of *c-Myc*^+^ (middle panel), and expression of *Cd34*^+^ (lower panel) in HSC clusters. (**B**) qRT-PCR analysis of *c-Myc*, *Cd34*, and *Id2* in purified HSCs. (**C**) Procedure to analyze FLT3^–^CD34^–^ HSPCs 2 weeks after deletion of *Id2* (left panel). Flow cytometry contour plots of *Id2^+/+^*and *Id2*^Δ/Δ^ LSK, FLT3, CD34 stained HSPCs (middle panel) and frequency (upper right panel) and total (lower right panel) LSK FLT3^–^CD34^–^ HSCs. (**D**) RNA-Seq procedure using *Id2^+/+^*and *Id2*^Δ/Δ^ HSCs, 2 weeks after *Id2* deletion. (**E**) Heatmap of differentially expressed genes (DEGs) between *Id2^+/+^* and *Id2*^Δ/Δ^ HSCs. (**F**) Summary of DEGs between *Id2^+/+^*and *Id2*^Δ/Δ^ HSCs. (**G**) GSEA of *Id2^+/+^* and *Id2*^Δ/Δ^ HSC RNA-Seq data sets. In **B** and **C**, data are presented as the mean ± SEM. Comparisons between the mean values of 2 groups were evaluated using an unpaired, 1-tailed tudent’s *t* test. *P ≤ 0.05, **P ≤ 0.01, and ***P ≤ 0.001.

To uncover the molecular mechanism or mechanisms that mediate loss of HSC quiescence in *Id2*^Δ/Δ^ HSCs, we performed single-cell RNA-Seq (scRNA-Seq) and RNA-Seq to compare the transcriptomes of *Id2^+/+^* and *Id2*^Δ/Δ^ HSCs purified from BMCs 2 weeks after pIpC treatment. First, scRNA-Seq revealed small numbers of contaminating neutrophils and RBCs, which were identified by the expression of lineage-specific genes and removed by cluster analysis, while the remaining HSCs were analyzed and visualized using the Loupe Cell Browser (Supplemental Figure 4, C and D). *Id2^+/+^* and *Id2*^Δ/Δ^ HSC gene expression analysis revealed 3 classes of HSCs, including HSCs (expressing Hlf, Cdkn1c, and Pml), activated HSCs (expressing Cd34, Sox4, Ccnb2), and platelet-biased HSCs (expressing *Pf4*, *Vwf*, *Cdk1*), based on previously established gene sets (35–39). Furthermore, we found that *Id2*^Δ/Δ^ HSCs were enriched for activated and platelet-biased HSCs, suggesting that *Id2*^Δ/Δ^ HSCs were more differentiated and resembled more aged HSCs (Figure 5A). In addition, *Id2*^Δ/Δ^ HSCs showed increased expression of cell-cycle genes including *c-Myc*, *Ccnd2*, *Ccnb1*, *Ccne1*, *Ccnd1*, and *Ccna2*, supporting the conclusion that *Id2*^Δ/Δ^ HSCs were actively cycling and proliferating compared with *Id2^+/+^* HSCs (Figure 5, A and B, and Supplemental Figure 4E). RNA expression levels of the HSC activation marker CD34 were increased in *Id2*^Δ/Δ^ HSCs compared with levels in HSCs from control mice (Figure 5, A and B). Immunophenotype analysis of HSCs 2 weeks after ablation of *Id2* confirmed that the frequency and number of LSK CD34^–^FLT3^–^ HSCs were reduced in *Id2*^Δ/Δ^ HSCs compared with *Id2^+/+^* HSCs (Figure 5C).

**Figure 6:**
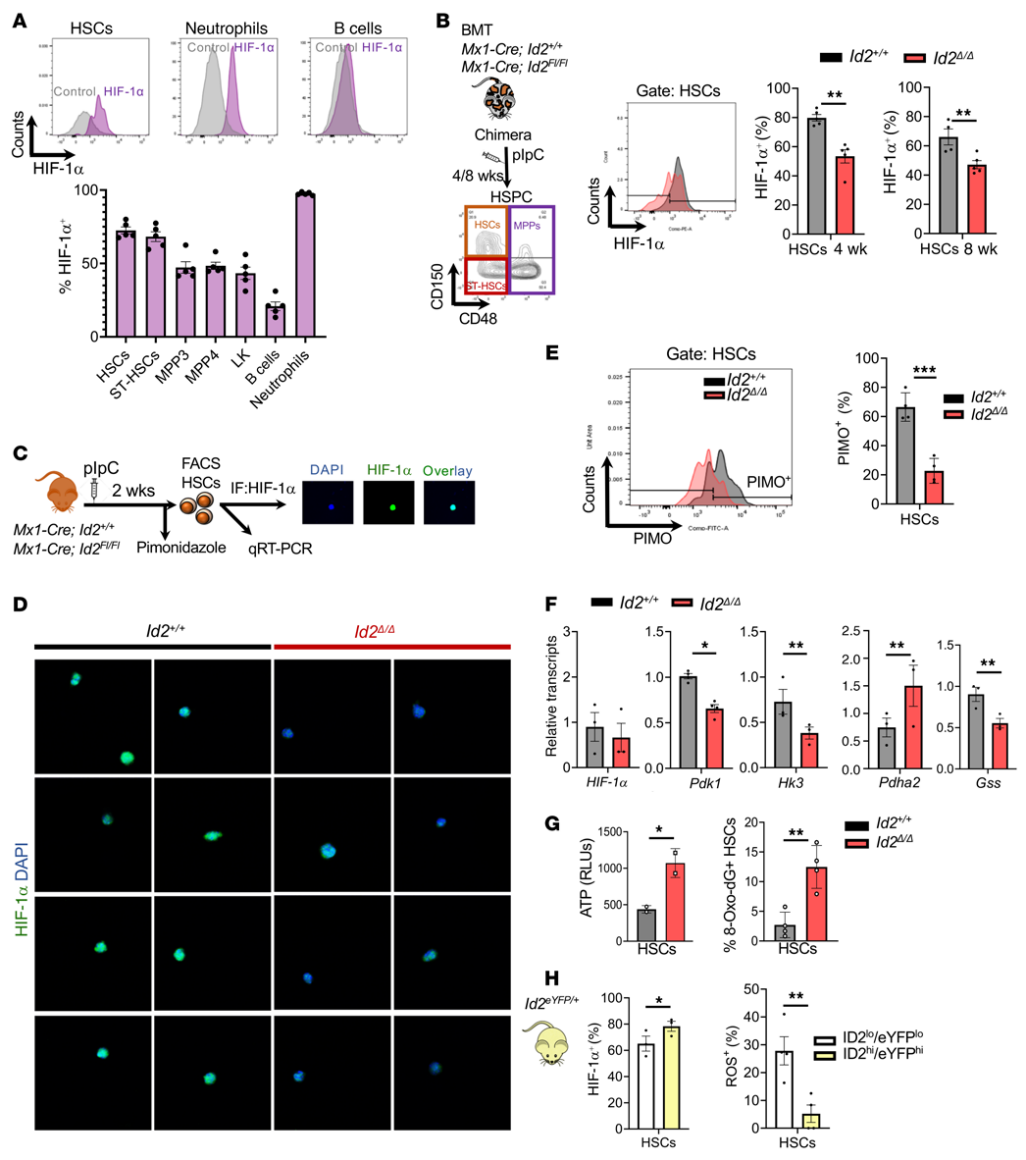
Loss of *Id2* results in decreased expression of HIF-1α and glycolytic target genes and hypoxic status in HSCs. (**A**) Analysis of HIF-1α expression in HSCs, neutrophils, and B cells by flow cytometry and the percentage of HIF-1α expression in HSPCs. (**B**) Flow cytometric analysis of HIF-1α expression in *Id2^+/+^* and *Id2*^Δ/Δ^ HSCs from chimeric mice, 4 and 8 weeks after *Id2* deletion. (**C**) Procedure to measure HIF-1α expression and hypoxic status (PIMO staining) of *Id2^+/+^* and *Id2*^Δ/Δ^ HSCs. (**D**) FACS-sorted *Id2^+/+^* and *Id2*^Δ/Δ^ HSCs were stained with antibodies that detect HIF-1α and imaged by confocal microscopy. (**E**) Pimonidazole levels in *Id2^+/+^* and *Id2*^Δ/Δ^ HSCs determined by flow cytometry. Original magnification, 40×. (**F**) Analysis of HIF-1α targets in *Id2^+/+^* and *Id2*^Δ/Δ^ HSCs, including genes related to glycolysis (*Pdk1*, *Hk3)*, oxidative phosphorylation (OXPHOS) (*Pdh1a2*), and ROS (*Gss*). (**G**) Estimation of ATP levels and oxidized DNA in *Id2^+/+^* and *Id2*^Δ/Δ^ HSCs. (**H**) HIF-1α expression and ROS levels in ID2^hi/lo^eYFP^hi/lo^ HSCs. In **B**, **E**, **F**, **G**, and **H**, data are presented as the mean ± SEM. Comparisons between the mean values of 2 groups were evaluated using an unpaired, 1-tailed Student’s *t* test. *P ≤ 0.05, **P ≤ 0.01, and ***P ≤ 0.001.

Mechanistically, it is unlikely that ID2 promotes HSC quiescence by regulating E proteins in these cells, since loss of ID gene function leads to increased E protein function and growth arrest (40–42). Therefore, to uncover the molecular pathways and genes that mediate HSC exhaustion in mice lacking *Id2*, we performed RNA-Seq to obtain a more in-depth transcriptome analysis of *Id2*^Δ/Δ^ and *Id2^+/+^* HSCs (Figure 5D). Gene set enrichment analysis (GSEA) of differentially expressed genes in RNA-Seq data sets revealed that *Id2*^Δ/Δ^ HSCs had increased expression of genes that regulate proliferation and growth (*Mcm10*, *Ccnb1*, *Ccnb2*, *Cdk1*), increased expression of oxidative phosphorylation pathway genes (*Cox10*, *Cyc1*, *Ppa2*), decreased expression of HIF-1α target genes (*Aldh2*, *Glut1*, *Hk3*), and decreased expression of HSC quiescence genes (*Atm*, *Atr*, *Foxo1*), suggesting that ID2 may affect the levels or function of HIF-1α (Figure 5, D–G, and Supplemental Figure 5A). Ingenuity Pathway Analysis (IPA) (QIAGEN) confirmed that *Id2*^Δ/Δ^ HSCs had increased expression of cell-cycle and oxidative phosphorylation pathway genes and decreased expression of HIF-1α signaling pathway genes (Supplemental Figure 5A). No difference in the levels of *Hif1a* RNA was observed in the RNA-Seq data or quantitative reverse transcription PCR (qRT-PCR) analysis of *Id2*^Δ/Δ^ and *Id2^+/+^* HSCs, whereas the expression of *Hif1a* target genes was decreased, suggesting that loss of *Id2* may affect the levels of HIF-1α protein (Figure 6F). Therefore, we examined the expression of HIF-1α in *Id2*^Δ/Δ^ and *Id2^+/+^* HSPCs by flow cytometry and confirmed that *Id2^+/+^* HSCs expressed high levels of HIF-1α, which was decreased in MPPs, Lin-c-Kit^+^ (LK) cells, and B cells but was highly expressed in neutrophils, as expected (Figure 6A and ref. 43). HIF-1α levels were significantly decreased in *Id2*^Δ/Δ^ HSCs and ST-HSCs 4 and 8 weeks after pIpC administration in chimeric mice compared with *Id2^+/+^* mouse HSCs, demonstrating that HSCs that lacked *Id2* had reduced HIF-1α expression (Figure 6B). Immunofluorescence analysis of FACS-purified HSCs confirmed that the levels of HIF-1α were markedly reduced in *Id2*^Δ/Δ^ HSCs compared with levels in *Id2^+/+^* HSCs (Figure 6, C and D). Thus, loss of *Id2* resulted in decreased HIF-1α levels, which could account for the increased proliferation and decreased quiescence of *Id2*^Δ/Δ^ HSCs, since mice that lack *Hif1a* have hematopoietic phenotypes that closely resemble that of *Id2*^Δ/Δ^ mice, including increased cycling, decreased quiescence, and increased susceptibility to 5-FU treatment (44).

Quiescent HSCs reside in the BM in a hypoxic cell state (44–46); therefore, we hypothesized that loss of *Id2* and *Hif1a* would reduce the number of hypoxic HSCs in vivo. We found that the levels of the hypoxic cell marker pimonidazole (PIMO) were significantly reduced in freshly isolated *Id2*^Δ/Δ^ HSCs compared with levels in *Id2^+/+^* HSCs (Figure 6E). Furthermore, reduced expression of HIF-1α in HSCs could activate and promote HSC proliferation, which is correlated with increased levels of mitochondrial activation, ROS generation, and DNA damage. Therefore, we measured the levels of tetramethylrhodamine methyl ester (TMRM), ROS, and γH2AX phosphorylation in *Id2^+/+^* and *Id2*^Δ/Δ^ HSCs from chimeric (4 and 8 weeks) and in *Mx1-Cre* *Id2^fl/fl^* mice after *Id2* deletion by HSPC analysis and flow cytometry. We found that *Id2*^Δ/Δ^ HSCs had increased levels of TMRM staining, suggesting increased mitochondrial activity compared with *Id2^+/+^* HSCs from chimeric mice (4 and 8 weeks after *Id2* deletion) and in conventional knockout mice (2 and 12 weeks after *Id2* deletion) (Supplemental Figure 5, B and C). We also observed increased ROS levels in *Id2*^Δ/Δ^ HSCs compared with levels in *Id2^+/+^* HSCs at the time we observed increased TMRM staining in chimeric mice and in *Mx1-Cre* *Id2^fl/fl^* mouse models (Supplemental Figure 5C). Finally, purified *Id2*^Δ/Δ^ HSCs show increased intracellular ATP content compared with *Id2^+/+^* HSCs, indicating that the *Id2*^Δ/Δ^ HSCs were more metabolically active (Figure 6G, left panel). *Id2*^Δ/Δ^ HSCs show increased levels of γH2AX phosphorylation, which was correlated with increased proliferation and DNA damage in chimeric mice 8 weeks after *Id2* deletion, and in *Mx1-Cre* *Id2^fl/fl^* mice 12 weeks after *Id2* deletion (Supplemental Figure 5C). Finally, we found that 8-Oxo-dG levels were increased on *Id2*^Δ/Δ^ HSCs compared with *Id2^+/+^* HSCs, confirming that the *Id2*^Δ/Δ^ HSCs had increased DNA damage (Figure 6G, right panel). Collectively, these results predict that HSCs with high levels of ID2 would express high levels of HIF-1α and low levels of ROS under steady-state conditions. Therefore, we compared HIF-1α expression and ROS levels in ID2^hi^eYFP^hi^ and ID2^lo^eYFP^lo^ HSCs in *Id2^eYFP/+^* reporter mice and found that ID2^hi^eYFP^hi^ HSCs had higher levels of HIF-1α and lower levels of ROS than did ID2^lo^eYFP^lo^ HSCs (Figure 6H). Taken together, loss of *Id2* and HIF-1α in HSCs promoted increased mitochondrial activation, ROS production, and DNA damage, resulting in decreased quiescence, which may have contributed to the observed decrease in HSC numbers, loss of HSC function, and HSC exhaustion.

### HIF-1α promotes quiescence and rescues HSC exhaustion of Id2^Δ/Δ^ HSCs in vitro and in vivo.

**Figure 7:**
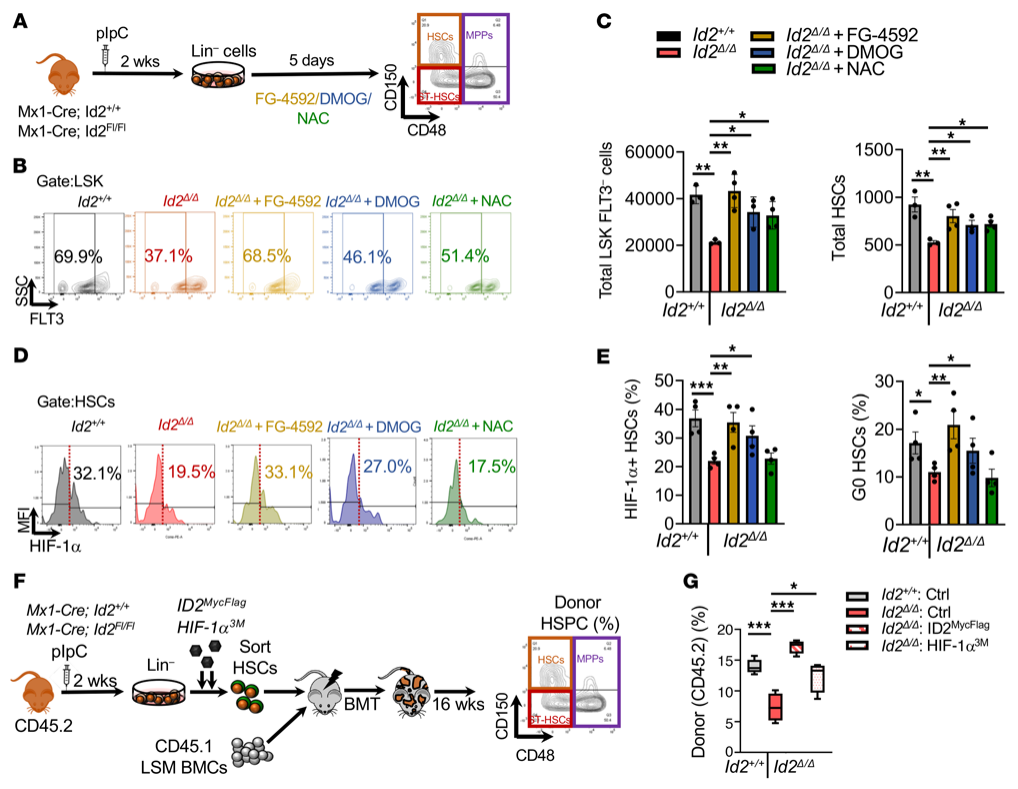
Stabilization of HIF-1α in *Id2^Δ/Δ^* HSCs rescues HSC numbers and promotes quiescence in vitro. (**A**) Procedure to rescue *Id2*^Δ/Δ^ loss of function using FG-4592 and DMOG to stabilize HIF-1α expression and NAC to reduce ROS in stem cell expansion assays. (**B**) Frequency of LSK FLT3^–^ cells in *Id2^+/+^* and *Id2*^Δ/Δ^ cultures treated with FG-4592, NAC, or DMOG. (**C**) Total number of LSK FLT3^–^ cells and HSCs following the indicated treatment in stem cell expansion assays. (**D**) Flow cytometric analysis of HIF-1α expression in *Id2^+/+^* and *Id2*^Δ/Δ^ HSCs after the indicated treatments. (**E**) Quantitation of HIF-1α expression (left) and quiescence (right) in HSCs after treatment with FG-4592, NAC, or DMOG. (**F** and **G**) Summary of the procedure to rescue HSC function in *Id2*^Δ/Δ^ HSCs with control, ID2^MycFlag^, and HIF1α^3M^ lentiviral expression vectors (left). Twenty transduced HSCs were combined with 50,000 LSM BMCs and transplanted into irradiated recipient mice and then analyzed for donor reconstitution after 16 weeks (right). In **C** and **E**, data are presented as the mean ± SEM. Comparisons between mean values of 2 groups were evaluated using an unpaired, 1-tailed Student’s *t* test, and 2-way ANOVA with Dunnett’s correction was used for multiple testing. In **F**, the center line indicates the median, and the box represents the 25th and 75th percentiles. *P ≤ 0.05, **P ≤ 0.01, and ***P ≤ 0.001.

To determine whether increased activation, proliferation, and exhaustion of *Id2*^Δ/Δ^ HSCs is due to reduced HIF-1α expression and increased ROS production, we treated expansion cultures with the proline hydroxylase (PHD) inhibitors FG-4592 and DMOG to stabilize HIF-1α expression, and *N*-acetyl cysteine (NAC) to reduce ROS levels (Figure 7A). As expected, the frequency and number of *Id2*^Δ/Δ^ FLT3^–^ progenitors and LSK FLT3^–^CD150^+^CD48^–^ HSCs were significantly reduced in expansion cultures after 5 days compared with *Id2^+/+^* HSCs; however, FLT3^–^ progenitors and HSC numbers were partially rescued in *Id2*^Δ/Δ^ cultures treated with FG-4592 and DMOG (Figure 7, B and C). Rescue of *Id2*^Δ/Δ^ HSCs by FG-4592 and DMOG was correlated with increased HIF-1α expression (Figure 7E, left panel) and increased quiescence (Figure 7E, right panel). Further, NAC treatment rescued loss of *Id2*^Δ/Δ^ HSCs in expansion assays but did not affect the levels of HIF-1α or cell growth (Figure 7, B–E). HSC numbers in control expansion and maintenance cultures were not significantly affected by FG-4592, DMOG, or NAC treatment, although there was a trend toward increased HSCs in expansion cultures treated with FG-4592, suggesting that these drugs did not significantly affect *Id2^+/+^* HSCs in these assays (Supplemental Figure 6, A and B). Finally, we transduced *Id2*^Δ/Δ^ HSCs with lentiviral vectors that expressed mutated HIF-1α (HIF-1α^3M^), preventing HIF-1α degradation, or with ID2 and GFP proteins, and determined whether the *Id2*^Δ/Δ^ HSCs were protected from proliferative exhaustion by analyzing their function in competitive repopulation assays (Figure 7, F and G). As predicted, *Id2*^Δ/Δ^ HSCs showed reduced donor reconstitution compared with *Id2^+/+^* HSCs 16 weeks after BMT, and enforced expression of ID2 and HIF-1α^3M^ in *Id2*^Δ/Δ^ HSCs increased donor reconstitution, demonstrating that *Id2* and *Hif1a* rescued the loss of HSC function and repopulation potential of *Id2*^Δ/Δ^ HSCs. Taken together, these results provide evidence that loss of expression of ID2 and HIF-1α and increased ROS levels in *Id2*^Δ/Δ^ HSCs promoted HSC cycling and differentiation that led to HSC exhaustion in vitro.

**Figure 8:**
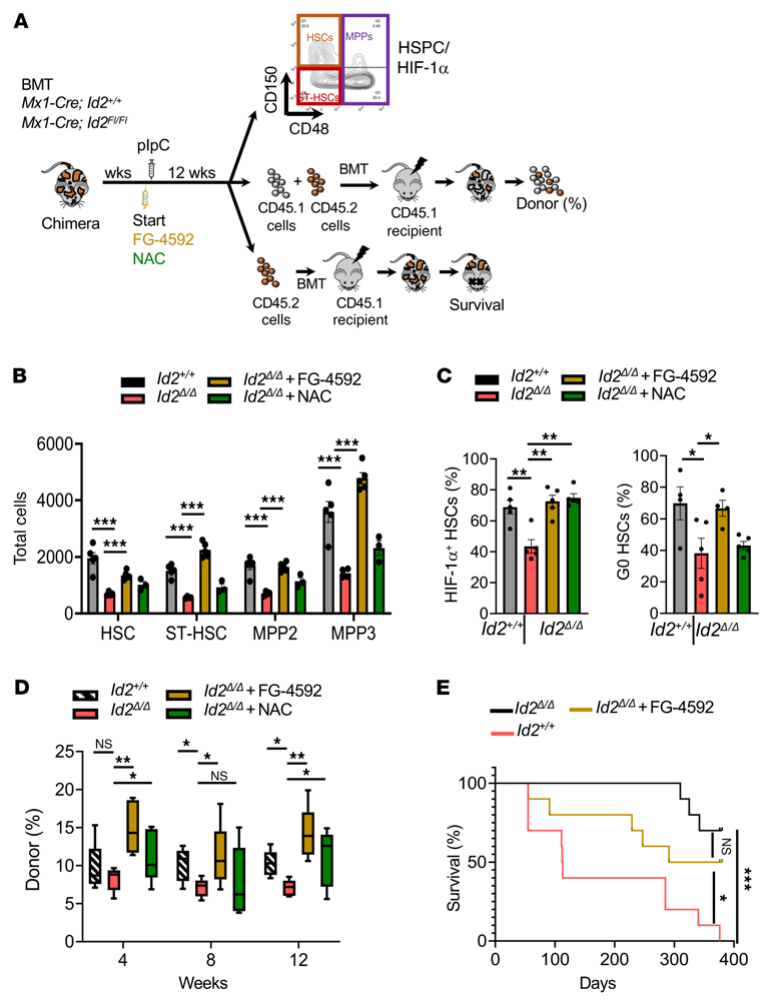
Stabilization of HIF-1α in *Id2^Δ/Δ^* HSCs rescues HSC numbers and promotes quiescence in vivo. (**A**) Procedure to measure the function of *Id2*^Δ/Δ^ HSCs in chimeric mice following treatment with FG-4592 or NAC. Mice were injected i.p. with 10 mg/kg FG-4592, or s.c. with 100 mg/kg NAC, every other day for 4, 8, or 12 weeks. (**B**) Total HSCs, ST-HSCs, and MPPs in chimeric mice following 12 weeks of treatment with FG-4592 or NAC. (**C**) Expression of HIF-1α in HSCs (left) and quiescence in HSCs (right) from chimeric mice following 12 weeks of treatment with FG-4592 or NAC. (**D**) Donor reconstitution of PBCs from mice competitively transplanted with BMCs from chimeric mice treated with FG-4592 or NAC for 12 weeks. (**E**) Survival of mice transplanted with BMCs from treated chimeric mice. In **B**, **C**, and **D**, data are presented as the mean ± SEM. Comparisons between mean values of 2 groups were evaluated using an unpaired, 1-tailed Student’s *t* test. A 2-way ANOVA with Dunnett’s correction was used for multiple means testing. In **D**, the center line indicates the median, and the box represents the 25th and 75th percentiles. Kaplan-Meier survival studies were analyzed using Wilcoxon’s signed-rank test. *P ≤ 0.05, **P ≤ 0.01, and ***P ≤ 0.001.

To determine whether reduced HIF-1α expression in *Id2*^Δ/Δ^ mice mediates HSC exhaustion in vivo, we treated chimeric mice with FG-4592 to stabilize HIF-1α expression and with NAC to reduce ROS levels, for 12 weeks (Figure 8A). As anticipated, HSC numbers were reduced in *Id2*^Δ/Δ^ chimeric mice compared with numbers in *Id2^+/+^* HSCs 12 weeks after ablation of *Id2*; however, loss of immunophenotypic HSCs in *Id2*^Δ/Δ^ chimeric mice was rescued by treating the mice with FG-4592 or NAC (Figure 8B). The rescue of HSCs in *Id2*^Δ/Δ^ chimeric mice treated with FG-4592 was correlated with increased expression of HIF-1α (Figure 8C). Finally, we confirmed that functional HSCs were rescued in *Id2*^Δ/Δ^ chimeric mice treated with FG-4592 or NAC in competitive repopulation assays. Specifically, BMCs from 12-week-old *Id2*^Δ/Δ^ chimeric mice showed reduced donor reconstitution potential compared with BMCs from *Id2^+/+^* chimeric mice in competitive BMT experiments, and donor reconstitution potential was rescued in *Id2*^Δ/Δ^ HSCs treated with FG-4592 or NAC (Figure 8D). Furthermore, mice transplanted with FG-4592–treated *Id2*^Δ/Δ^ HSCs show increased survival compared with control-treated *Id2*^Δ/Δ^ HSCs, confirming that FG-4592 rescued the donor reconstitution potential of *Id2*^Δ/Δ^ HSCs (Figure 8E). These results provide evidence that reduced HIF-1α expression in *Id2*^Δ/Δ^ HSCs mediated the loss of HSC numbers and function in vivo.

### HIF-1α promotes Id2 expression to maintain HSCs in vitro.

**Figure 9:**
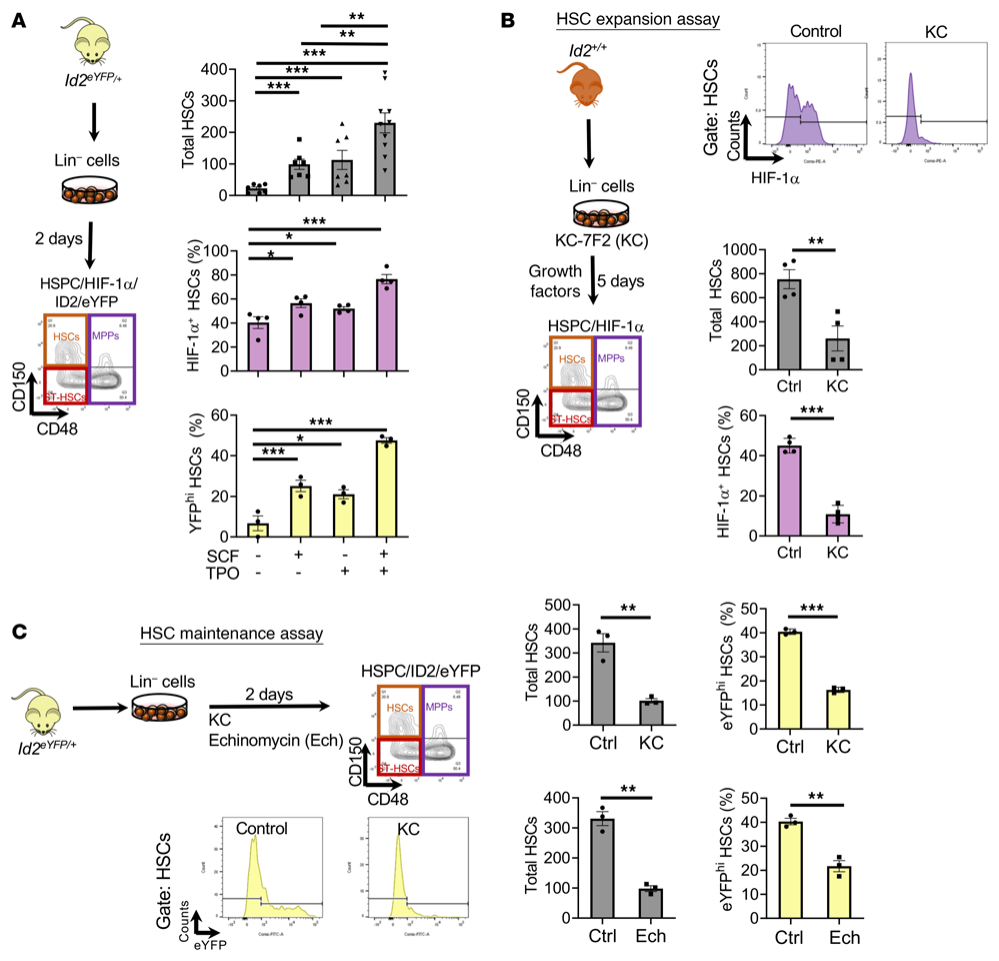
SCF and TPO promote ID2 and HIF-1α expression in a feed-forward loop to maintain HSCs. (**A**) Procedure to analyze HIF-1α and ID2/eYFP expression in maintenance cultures with SCF, TPO, or SCF plus TPO. Quantitation of HSCs (top), HIF-1α expression in HSCs (middle), and ID2/eYFP expression in HSCs after 2 days of culturing (bottom). (**B**) Procedure to analyze HIF-1α expression in HSPCs in Lin^–^ expansion cultures 5 days after treatment with KC-7F2 (KC). Flow cytometric histogram plots of HIF-1α expression in HSCs (top), total HSCs in culture (middle), and HIF-1α expression in HSCs (bottom). (**C**) Procedure to evaluate ID2/eYFP expression in HSCs in maintenance cultures of Lin^–^ ID2^eYFP^ cells after 2 days of treatment with KC-7F2 and echinomycin (Ech). Flow cytometric histogram plots below the procedure schema show ID2/eYFP expression in HSCs in control and KC-7F2–treated cultures. Graphs on the right show quantitation of HSCs and ID2/eYFP expression in KC-7F2–treated cultures (top) and quantitation of HSCs and ID2/eYFP expression in echinomycin-treated cultures (bottom). In **A**–**C**, data are presented as the mean ± SEM. Comparisons between mean values of 2 groups were evaluated using an unpaired, 1-tailed Student’s *t* test, and 1-way ANOVA with Dunnett’s correction was used for multiple means testing in **A**. *P ≤ 0.05, and **P ≤ 0.01, and ***P ≤ 0.001. Ctrl, control.

Previous reports demonstrated that *Id2* is a direct transcriptional target of HIF-1α in neuroblastoma cells, suggesting that HIF-1α and ID2 may function in a feed-forward loop in HSCs (47, 48). Therefore, we examined whether chemical inhibitors of HIF-1α reduce ID2 expression and phenocopy *Id2*^Δ/Δ^ HSC exhaustion using Lin^–^ cells from *Id2^eYFP/+^* reporter mice. First, since stem cell factor (SCF) and thrombopoietin (TPO) maintain HSCs in vitro and in vivo and promote *Hif1a* mRNA expression in HSCs, we speculated that SCF and TPO may increase *Id2* in HSCs via *Hif1a* expression (44, 49, 50). As expected, HSC survival was increased in the presence of SCF or TPO, and maximally by the combination of SCF and TPO, in maintenance cultures of Lin^–^ BMCs from *Id2^eYFP/+^* mice after 48 hours (Figure 9A, upper panel). In addition, SCF plus TPO promoted maximal HIF-1α and ID2^eYFP^ expression in HSCs compared with SCF or TPO alone (Figure 9A, middle and lower panels). We observed a marked decrease in ID2/eYFP expression in HSCs in the maintenance cultures treated with KC-7F2 (inhibits HIF-1α translation) and echinomycin (inhibits HIF-1α transcriptional activity) after 48 hours compared with control-treated cultures (Figure 9C). Furthermore, the total number of HSCs decreased in these cultures from approximately 350 to approximately 100 HSCs with either inhibitor (Figure 9C). Similarly, KC-7F2 treatment of Lin^–^ BMCs in expansion cultures resulted in a 76% reduction of HIF-1α expression in HSCs and a 65.5% reduction of total HSCs (Figure 9B). Thus, inhibition of HIF-1α expression or activity resulted in reduced ID2 transcription and ID2/eYFP levels and HSC exhaustion in vitro.

Since HIF-1α is stabilized under hypoxic conditions, we speculated that incubating Lin^–^ BMCs from *Id2^eYFP/+^* reporter mice in low O_2_ would increase HIF-1α and ID2 expression. HIF-1α levels were increased in Lin^–^ cells cultured under low O_2_ conditions compared with normoxia, and the percentage of Lin^–^ cells that coexpressed ID2/eYFP and HIF-1α were increased under low O_2_ conditions (Supplemental Figure 6, C and D). Similarly, HIF-1α and ID2 levels were increased in the erythroid myeloid lymphoid (EML) stem cell line, cultured under hypoxic conditions (Supplemental Figure 6E), and in EML cells treated with DMOG, FG-4592, or CoCl_2_, (which stabilize HIF-1α expression (Supplemental Figure 6E).

**Figure 10:**
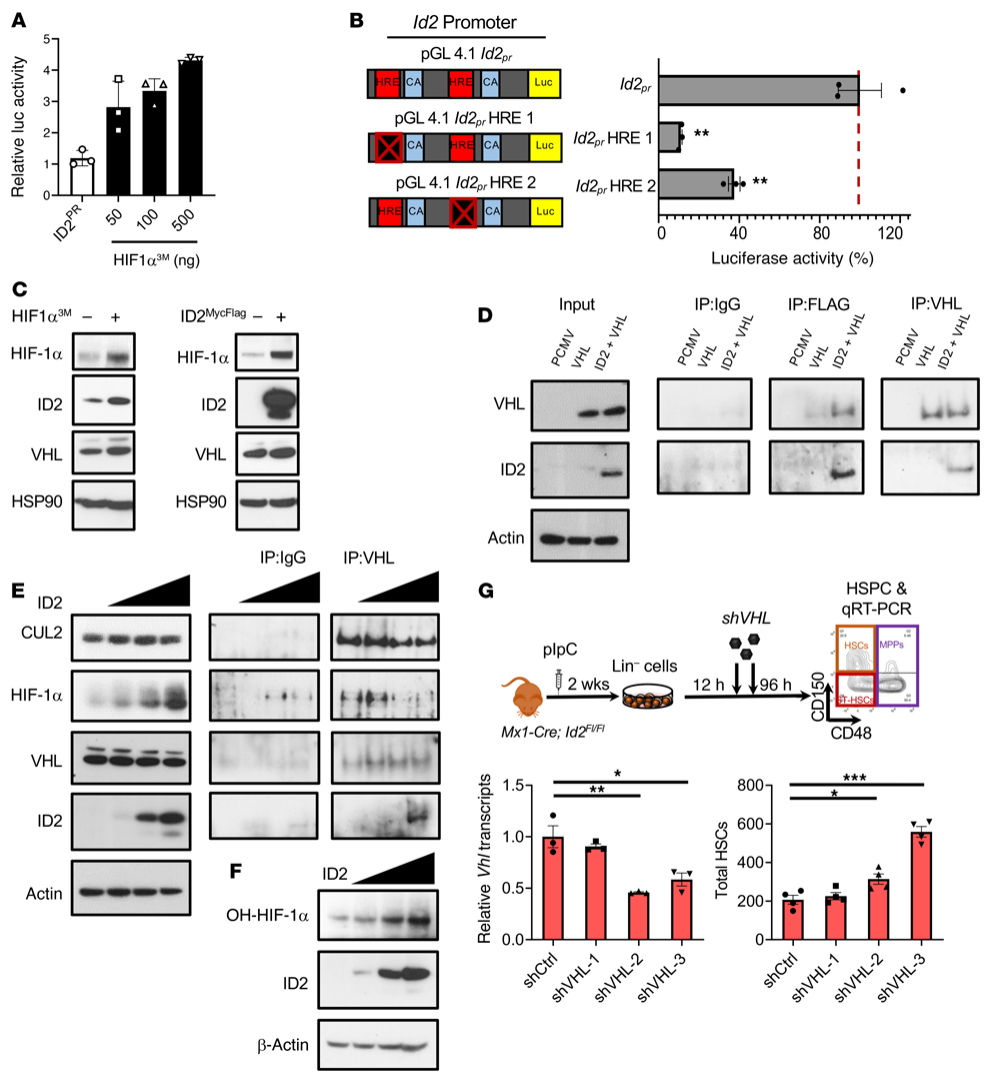
HIF-1α regulates *Id2* promoter activity, and ID2 stabilizes HIF-1α and promotes HSC survival via the VHL complex in vitro. (**A**) Luciferase (Luc) activity of pGL4.1 *Id2_promoter_* in HEK293 cells transfected with pcDNA3.1 or pcDNA3.1 HIF-1α^3M^ (HIF-1α). (**B**) Schematic of the cloned *Id2_promoter_* with identified HRE-CACA boxes for luciferase reporter assays (left) and luciferase activity of the pGL4.1 *Id2_promoter_* with mutated HRE boxes transfected with pcDNA3.1 HIF-1α^3M^ (right). (**C**) Western blot analysis of HIF-1α, ID2, VHL, and HSP90 in lysates from HEK293 cells transfected with either pcDNA3.1 or pcDNA3.1 mutated HIF-1α^3M^ or PCMV or PCMV-ID2^MycFlag^. (**D**) Western blot analysis of VHL, ID2, and β-actin and IP analysis of ID2^MycFlag^ and VHL^HA^ in HEK293 lysates transfected with VHL or ID2 constructs. (**E**) Western blot analysis of CUL2, HIF-1α, VHL, ID2, and β-actin and IP analysis of VHL in HEK293 lysates transfected with increasing amounts of ID2^MycFlag^. (**F**) Western blot analysis of hydroxylated HIF-1α (OH–HIF-1α), ID2, and β-actin in HEK293 cell lysates transfected with increasing amounts of ID2. (**G**) Procedure to measure HSC expansion after knockdown of *Vhl* expression in *Id2*^Δ/Δ^ Lin^–^ cells using lentiviral control shRNA (shCtrl) and shRNA-VHL (shVHL-1, -2, -3) vectors. Graphs show the expression of *Vhl* transcripts in knockdown Lin^–^ cells and total HSCs in culture. In **B** and **G**, data are presented as the mean ± SEM. Comparisons between mean values of 2 groups were evaluated using an unpaired, 1-tailed Student’s *t* test, and 1-way ANOVA with Dunnett’s correction was used for multiple means testing. *P ≤ 0.05, **P ≤ 0.01, and ***P ≤ 0.001.

To determine whether HIF-1α directly regulates *Id2* expression, we examined the *Id2* promoter and identified 2 canonical HREs adjacent to 3′ CACA boxes, suggesting that *Id2* may be a direct target of HIF-1α (Figure 10B and refs. 51, 52). We found that HIF-1α induced *Id2* promoter reporter activity in transfected HEK293 cells (Figure 10A). Furthermore, mutating the HRE1 and HRE2 sites significantly reduced HIF-1α–induced *Id2* promoter reporter activity (Figure 10B). These data suggest that *Id2* is a direct target of HIF-1α.

### Id2 stabilizes HIF-1α protein expression in HSCs by preventing ubiquitin-mediated proteasomal degradation of HIF-1α in HSCs.

We provide evidence that ablation of *Id2* resulted in reduced HIF-1α expression in HSCs without affecting *Hif1a* RNA expression, which suggests that *Id2* directly or indirectly stabilized HIF-1α in HSCs. Enforced expression of *Hif1a* promotes ID2 expression in HEK293 cells (Figure 10C, left panel, and Supplemental Figure 6F). Enforced expression of *Id2* promoted HIF-1α expression in HEK293 cells (Figure 9C, right panel), and increased luciferase activity of a cotransfected 3X HRE luciferase reporter plasmid, confirming that ID2 could increase HIF-1α expression and function (Supplemental Figure 6G). In addition, ID2 induced HIF-1α expression in a dose-dependent manor in TetI-ID2-U2OS, without affecting the expression of HIF-2α, aryl hydrocarbon receptor nuclear translocator (ARNT, also known as HIF-1β), or VHL proteins (Supplemental Figure 6H). Furthermore, ID2 induced HIF-1α expression in human hematopoietic AML cell lines in a dose-dependent manor (Supplemental Figure 6I). These data provide evidence that ID2 specifically stabilized HIF-1α, and not HIF-2α or HIF-1β.

Since ID2 can prevent proteasomal degradation of HIF-2α in glioblastoma cells by binding to VHL protein present in the Cullin ring E3 ubiquitin ligase (CRL) complex (53), we examined whether ID2 could inhibit HIF-1α degradation in HSCs. First, we confirmed that ID2 was present in VHL IP complexes and that VHL was present in ID2 IP complexes in HEK293 cell lysates (Figure 10D). Furthermore, we demonstrated that HIF-1α was present in the VHL complex and was decreased in VHL complexes with increasing concentrations of ID2 (Figure 10E). In addition, we show that cullin 2 (CUL2) was also decreased in the VHL complexes with increasing concentrations of ID2 (Figure 10E). Collectively, these findings support the conclusion that ID2 stabilized HIF-1α expression by binding VHL and preventing HIF-1α and CUL2 from binding the VHL complex. Finally, we show that the levels of hydroxylated HIF-1α were increased in cell lysates with increasing concentrations of ID2, confirming that HIF-1α was stabilized,and that ID2 did not interfere with the ability of PHD to hydroxylate HIF-1α, which is required for HIF-1α to bind and associate with VHL (Figure 10F).

To confirm that the HSC rescue in *Id2*^Δ/Δ^ expansion cultures with PHD inhibitors was through stabilization of HIF-1α via the VHL complex, we knocked down VHL expression in expansion cultures and determined HSC numbers. We found that VHL expression was reduced in Lin^–^ *Id2*^Δ/Δ^ BMCs treated with shRNA-VHL lentiviral vectors compared with the control shRNA, which resulted in increased numbers of HSCs after 4 days (shVHL-2, shVHL-3; Figure 10G). Furthermore, knockdown of VHL expression increased HIF-1α expression in HSCs in *Id2*^Δ/Δ^ expansion cultures (shVHL-2, shVHL-3; Supplemental Figure 6J). These results confirm that stabilization of HIF-1α via the VHL complex in *Id2*^Δ/Δ^ expansion cultures rescued the reduction in HSC numbers in vitro.

## Discussion

ID2 is a critical transcriptional regulator of hematopoietic lineage cell fate and development that are required for proper lymphoid, NK, and DC development by inhibiting E protein transcriptional activity (24, 26, 28, 30, 54, 55). In this study, we provide evidence that ID2 regulated HSC quiescence and function by stabilizing HIF-1α and that ablation of *Id2* in HSCs resulted in HSC activation, differentiation, and eventual exhaustion. The expression of HIF-1α was significantly reduced in *Id2*^Δ/Δ^ HSCs, and enforced expression of either ID2 or HIF-1α in *Id2*^Δ/Δ^ HSCs rescued HSC quiescence and prevented exhaustion. Finally, HIF-1α directly promoted *Id2* expression, suggesting that ID2 and HIF-1α enforced each other’s expression in a positive feedback loop to maintain HSCs.

The expression of HIF-1α was reduced in *Id2*^Δ/Δ^ HSCs, while HIF-2α and *Hif1a* transcripts were unaffected. HIF-1α target genes that promote glycolysis were decreased in *Id2*^Δ/Δ^ HSCs, while mitochondrial activation (TMRM), oxidative phosphorylation, and ROS production were increased, suggesting that HSCs shifted from glycolysis to oxidative metabolism after loss of *Id2* (56). In support of this observation, the ROS scavenger NAC rescued HSC quiescence and the function of *Id2*^Δ/Δ^ HSCs in vitro and in vivo. Furthermore, chemical stabilization or enforced HIF-1α expression in *Id2*^Δ/Δ^ HSCs promoted quiescence, rescued HSC numbers, and restored HSC function. *Id2*^Δ/Δ^ and *Hif1a*^Δ/Δ^ HSCs shared hematopoietic phenotypes including increased cell cycling, sensitivity to genotoxic stress, and decreased HSC repopulation potential, supporting the notion that ID2 maintains HSCs by increasing HIF-1α expression and its downstream targets.

We demonstrated that ID2 regulated HSC quiescence, in part by stabilizing HIF-1α; however, there is some debate in the literature about whether HIF-1α is required for HSC maintenance. Specifically, Takubo et al. reported that loss of *Hif1a* in HSCs resulted in increased proliferation and exhaustion when subjected to hematopoietic stress (44), whereas Vukovic et al. found that HIF-1α was dispensable for HSC maintenance and self-renewal (57). In related studies, the authors provide evidence that Meis1 promoted *Hif1a* expression and that *Meis1^–/–^* mice showed reduced *Hif1a* expression, which resulted in increased mitochondrial metabolism, ROS production, and loss of HSC function (58). Also, HSCs that lack the HIF-1α target genes *Pdk2* and *Pdk4* (*Pdk2/4*), which suppress the influx of glycolytic metabolites into the TCA cycle, show decreased quiescence and HSC function (59). Furthermore, overexpression of *Pdk2/4* rescues the hematopoietic defects observed in *Hif1a*^Δ/Δ^ mice, indicating that PDK2 and PDK4 act downstream of HIF-1α to promote glycolysis and HSC quiescence. Moreover, HIF-1α expression is increased in *VHL*^Δ/+^ and *Phd2*^Δ/Δ^ mice, which results in increased HSC self-renewal and decreased repopulation potential (44). Collectively, these studies provide evidence that HIF-1α is required to regulate normal HSC quiescence and proliferation.

Mechanistically, we show that ID2 stabilized HIF-1α by binding to VHL and interfering with ubiquitin-mediated proteasomal degradation. Similarly, ID2 binds to VHL in glioblastoma tumor cells and disrupts the binding of CUL2 to the VHL–elongin B–elongin C–CUL2 (VCB-CUL2) complex, which prevents ubiquitin-mediated proteasomal degradation of another HIF-1 family member, HIF-2α, and promotes the survival of glioblastoma stem cells (GSCs) (53). We show here that ID2 stabilized the expression of HIF-1α rather than HIF-2α in hematopoietic cells, inhibits the binding of HIF-1α to VHL, and displaced CUL2 from VHL, suggesting a similar molecular mechanism of ID2 action in HSCs and GSCs. Knockdown of VHL expression in *Id2*^Δ/Δ^ HSCs rescued HSC quiescence and function, supporting the conclusion that dysfunctional HIF-1α regulation significantly contributed to HSC exhaustion in *Id2*^Δ/Δ^ mice. While our data demonstrate that ID2 promoted HSC quiescence by stabilizing HIF-1α *Id2*^Δ/Δ^ HSCs, RNA-Seq data showed decreased expression of the HIF-1α targets *p57* and *Foxo*, as well as *c-Kit*, *Atm*, and *c-Mpl*, which may represent ID2-mediated molecular mechanism(s) of HSC quiescence not regulated by HIF-1α and could explain, in part, the hematopoietic phenotypes of *Id2*^Δ/Δ^ mice (60–65).

SCF and TPO are cytokines produced by the HME that promote HSC survival and *Hif1a* transcription in HSCs (49, 50, 66–68). While HIF-1α levels are normally regulated by tissue oxygen levels and are increased under hypoxic conditions (69), we demonstrate that ID2 could increase HIF-1α expression by interfering with the VHL complex under normoxic conditions. Since HIF-1α regulates *Id2* expression in promoter reporter assays, we speculated that a positive feedback loop exists between ID2 and HIF-1α. In support of this hypothesis, we show that increasing either SCF or TPO in cultures promoted HSC survival as well as HIF-1α and ID2/eYFP expression, and that together, SCF plus TPO promoted maximum HIF-1α and ID2/eYFP expression and survival of HSCs under normoxic conditions. Furthermore, chemical inhibition of HIF-1α in vitro reduced ID2/eYFP expression and reduced the number of surviving HSCs. Finally, stabilizing HIF-1α expression in EML cells resulted in increased ID2 levels in a dose-dependent manor. Collectively, these findings suggest that cytokines produced in the HME and low O_2_ levels initiated a positive feedback loop between HIF-1α and ID2 to promote HSC quiescence. Since SCF and ID2 promoted and stabilize HIF-1α expression under normoxic conditions, this may represent a molecular pathway for stabilizing HIF-1α expression and promoting HSC quiescence and survival under low or high tissue O_2_ conditions (50, 70). Thus, it will be important to further investigate and identify pathways other than that of HIF-1α that promote ID2 expression in HSCs. In addition, the collection and processing of BM at low O_2_ (3%) in vitro significantly improves HSC recovery by mitigating the effects of extraphysiological oxygen shock stress (EPHOSS), in part, by increasing HIF-1α levels and reducing ROS production (71–73).

Our results demonstrate that ID1 and ID2 had distinct, nonoverlapping functions in HSCs. ID1 is expressed in HSCs during proliferative stress including during BMT, genotoxic/inflammatory stress, and aging, where it functions by regulating CDKI expression via E proteins (20). In comparison, ID2 is constitutively expressed in HSCs and promoted quiescence, in part by stabilizing HIF-1α expression. These results suggest that the expression of ID1 and ID2 were inversely correlated in quiescent and proliferating HSCs. We found that ID2^hi^eYFP^hi^ HSCs were significantly more quiescent than ID2^lo^eYFP^lo^ cells under homeostasis and that ID2/eYFP was silenced in HSCs after proliferative stress, including genotoxic and inflammatory stress and BMT. Specifically, ID2^hi^eYFP^hi^ quiescent HSCs were enriched in BMCs 2 days after the administration of 5-FU, when cycling HSPCs were eliminated, after which the expression of ID2/eYFP in HSCs declined as HSCs proliferated to repopulate the host. Subsequently, ID2/eYFP increased in HSCs as they reentered quiescence. Similarly, we found that expression of ID2/eYFP was reduced in HSCs 1 day after BMT or LPS treatment, when HSCs were induced to proliferate, after which expression of ID2/eYFP recovered in HSCs as they repopulated the host and returned to quiescence. In comparison, ID1/GFP expression was increased in HSCs after BMT and inflammatory stress, when HSCs were signaled to proliferate (20). Thus, quiescent HSCs expressed low levels of ID1 and high levels of ID2, whereas HSCs induced to proliferate after stress expressed high levels of ID1 and low levels of ID2. While proinflammatory cytokines produced by cells in the HME after BM conditioning induced ID1 in HSCs, the precise mechanism or mechanisms that downregulate ID2 expression in HSCs after BMT or genotoxic stress are currently not known. Others have also observed an inverse correlation in ID1 and ID2 expression in patients’ chronic myelogenous leukemia (CML) and blast crisis cells and in nonhematopoietic tissue, where loss of ID2 expression and overexpression of ID1 promote colon and breast cancer (11, 74–76).

Ablation of *Id2* in HSCs results in increased proliferation, suggesting that ID2 may function as a tumor suppressor; however, we did not monitor the *Id2*^Δ/Δ^ mice in these experiments beyond 12–16 weeks, when hematopoietic malignancies might develop. Interestingly, Ko et al. showed that mice transplanted with *Id2^–/–^* fetal liver cells developed leukocytosis after 6 months that resembled a myeloproliferative disorder and that overexpression of ID2 delays the onset *Bcr-Abl*–induced CML in vivo (77). In addition, loss of *Id2* expression is associated with increased MLL-AF9–induced leukemia (78). Together, these results suggest that ID2 may function as a tumor suppressor in hematopoietic malignancies. In addition, mice that lack *Id2* develop intestinal adenomas and show a hyperproliferation of colon stem cells during embryonic development due to increased Wnt/β-catenin signaling, suggesting that ID2 may function as a tumor suppressor in other cell types (79, 80). Further studies are needed to determine whether ID2 is expressed in leukemic stem cells and regulates their quiescence and survival.

Overall, maintaining stem cell quiescence during periods of inflammatory, proliferative, and BMT stress is essential for preventing HSC exhaustion. The data show that ID2 is expressed in steady-state HSCs and silenced during periods of proliferative stress, suggesting that *Id2* must be downregulated to repopulate blood and BM lineages. Loss of *Id2* results in depressed HIF-1α expression and HSC exhaustion, thus ID2-HIF-1α–expressing HSCs are essential for HSC maintenance during periods of stress and recovery. HSCs expressing ID2 and HIF-1α are enriched for cells with enhanced serial repopulation potential. Thus, preserving ID2 and HIF-1α expression could have therapeutic potential to maintain HSCs under conditions of hematopoietic stress by balancing HSC quiescence and proliferation/differentiation during BMT in vivo, and to maintain HSC function and prevent differentiation and exhaustion during gene editing, gene therapy, and stem cell expansion in vitro.

## Methods

### Mice.

*Id2*-floxed (*Id2^fl^*) mice were generated by inserting PGK-Neo between exons 2 and 3 with loxP sites introduced upstream of exon 1 and between exons 2 and 3. Integration of the loxP PGK-neo cassette was confirmed by southern blotting. Mice were generated using the CCR Gene Targeting Facility at the NCI, NIH. Mice were backcrossed with C57BL/6 mice (Charles River Laboratories) at least 5 times before use. *Id2^fl/fl^* mice were crossed with *R26-M2rtTA* *TetOP-H2B-GFP* mice (81) obtained from The Jackson Laboratory and *Mx1-Cre* mice (82). *Id2^eYFP/+^* mice were generated by inserting a *eYFP PGK-Neo* downstream of the transcriptional start site using the CCR Gene Targeting Facility at the NCI (Supplemental Figure 1A). C57BL/6 Ly5.1 (CD45.1) mice were obtained from Charles River Laboratories. Eight- to 12-week-old female mice were used as recipients of BM transplants.

### BMT.

Chimeric mice were generated by transplanting 2.0 × 10^6^ *Mx1-Cre* *Id2^+/+^* or *Mx1-Cre* *ID2^fl/fl^* BM cells into γ-IR congenic CD45.1 recipient mice by tail vein injection, after which mice were treated with pIpC to ablate *Id2* expression. *Mx1-Cre* *Id2^+/+^* or *Mx1-Cre* mice were injected i.p. with 1 mg/mL pIpC (InvivoGen, tlrl-pic) according to weight (300 μL/20 g mouse) 6 weeks after transplantation. Mice that received γ-IR (10 Gy) were pretreated 1 week before and 2 weeks after transplantation with antibiotic-containing water (pH 2.5–3.0, 0.5 mg/mL amoxicillin, 0.17 mg/mL enrofloxacin). *Mx1-Cre* *Id2^+/+^* or *Mx1-Cre* *ID2^fl/fl^* (CD45.2) BMCs (1.0 × 10^6^) from chimeric mice were mixed with 1.0 × 10^6^ CD45.1 BMCs and transplanted into γ-IR CD45.1 recipient mice (primary recipients). BMCs (2 × 10^6^) from primary recipients were pooled and transplanted into secondary γ-IR recipient mice by tail vein injection after 16 weeks. Donor reconstitution was assessed in peripheral blood cells (PBCs) 4, 8, and 12 weeks after BMT and after 12 or 16 weeks in BM using flow cytometry. Twenty chimeric *Mx1-Cre* or *Mx1-Cre ID2^fl/fl^* mice and 5 noncompetitive mice were monitored for survival. Lethargic/sick mice were randomly selected, euthanized, and sent to the pathology/histology laboratory at the NCI to determine the cause of death. For stress BMT assays, BMCs were isolated from *Mx1-Cre* *Id2^+/+^* or *Mx1-Cre* *Id2^fl/fl^* mice 2–3 weeks after pIpC injection and then transplanted into lethally irradiated recipient mice. Alternatively, 2.0 × 10^6^ BMCs from *Id2^eYFP/+^* reporter mice were transplanted into lethally irradiated recipient mice. Marrow was harvested 1, 4, 7, and 28 days after BMT. Lymphocyte separation medium (LSM) BMCs were analyzed for HSPC markers and ID2/eYFP expression by flow cytometry. For HSC transplants, 25 LSK FLT3^–^CD150^+^CD48^–^ cells were sorted and mixed with either 2 × 10^5^ or 5 × 10^5^ C57BL/6 (CD45.1) BMCs and transplanted into γ-IR congenic CD45.1 mice.

### Hematopoietic stem and progenitor cell analysis by flow cytometry.

BMCs were harvested by flushing femurs and tibias with 1% BSA PBS using a 25 gauge needle and syringe and filtered through a 40 μm membrane. *Mx1-Cre* *Id2^+/+^* or *Mx1-Cre* *ID2^fl/fl^* BMCs or chimeric BMCs of animals transplanted with *Mx1-Cre* *Id2^+/+^* or *Mx1-Cre* *ID2^fl/fl^* BMCs were suspended in 1% BSA PBS. The light-density BMC fraction was isolated using lymphocyte separation media (LSM) (MO Biomedicals). PBCs collected from retroorbital bleeds were incubated with BD Pharm lysis buffer and washed in 1% BSA PBS solution. For lineage analysis, PBCs and BMCs were incubated with the following fluorochrome-conjugated monoclonal antibodies for lineage analysis: CD45.2 (clone 104), Mac-1 (M1/70), Gr-1 (RB6-8C5), B220 (RA3-6B2), CD4 (GK1.5), CD8 (53-6.7), CD71 (R17217), and Ter119 (Ter119). For HSPC analysis, purified LSM BMCs were treated with FcR blocking antibody (anti–mouse CD16/anti-CD32) and then incubated with fluorochrome-conjugated lineage markers (Mac-1, Gr-1, B220, TER119, CD4, CD8, and IL-7Rα); c-Kit (ACK2); Sca-1(D7); CD150 (mShad150); CD48 (HM48-1); CD34 (RAM34); FLT3 (A2F10); and anti-FcγRII/III (FcR). HSCs and long-term HSCs (LT-HSCs) were LSK FLT3^–^CD150^+^CD48^–^; ST-HSCs were LSK FLT3^–^CD150^–^CD48^–^; MPP3 cells were LSK FLT3^–^CD150^–^CD48^+^; and MPP2 cells were LSK FLT3^–^CD150^+^CD48^+^. All cells were incubated with antibodies in 1% BSA PBS for 30 minutes on ice and washed in buffer prior to analysis, and all of the antibodies used were purchased from BD Biosciences or eBioscience. For annexin V staining, BMCs stained for HSPC analysis were further incubated with annexin V–FITC (BD Biosciences) and analyzed immediately by flow cytometry. For cell-cycle/quiescence (Ki-67), HIF-1α analysis, and DNA damage assays (γH2AX), BMCs stained for HSPC analysis were fixed and permeabilized with Cytofix/Cytoperm buffer (BD Biosciences), followed by intracellular staining overnight with Ki-67–FITC (BD Biosciences, 556026); HIF-1α (R&D Systems, IC1935P); and γH2AX-PE (Cell Signaling Technology, 9718) antibodies. Similarly, for DNA oxidation (8-oxo-dG), BMCs were fixed and permeabilized, followed by treatment for 1 hour with DNase and subsequent staining for 8-oxo-dG (Trevigen, Thermo Fisher Scientific, 4354-MC-050). Cells were incubated with FxCycle Violet dye (Thermo Fisher Scientific) 2–4 hours before acquisition. The BD LSR II SORP, BD Symphony, and BD Fortessa were used for flow cytometric analysis and the BD FACSAria II for cell sorting. Data analysis was done using FlowJo software, version 10.4 or 10.7.1 (Tree Star). For PIMO staining, a dose of 75 mg/kg PIMO was administered 90 minutes before tissue harvesting. PIMO staining was performed according to the Hypoxyprobe kit instructions (Hypoxyprobe, HPI-4.3.11.3).

See Supplemental Methods for a detailed description of the additional experimental procedures. The RNA-Seq data are available in the NCBI’s Gene Expression Omnibus (GEO) database (GEO GSE198599).

## Author contributions

TS, KOG, SS, HMM, and KDK designed and conducted experiments. LS designed and conducted experiments and analyzed results. BLJ and JRK designed and conducted experiments, analyzed results, and wrote the manuscript.

## Supplementary Material

Supplemental data

## Figures and Tables

**Table 1:**
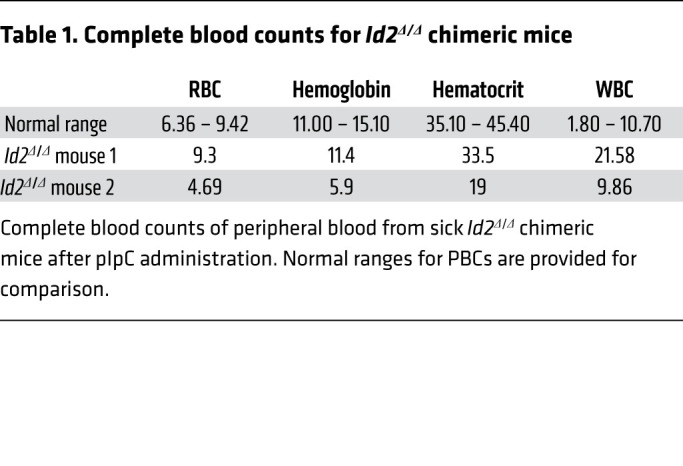
Complete blood counts for *Id2^Δ/Δ^* chimeric mice

## Version 1 07/01/2022

Electronic publication
