# Supplementary material for: ID2 and HIF-1α collaborate to protect quiescent hematopoietic stem cells from activation, differentiation, and exhaustion
Source: J Clin Invest. 2022 Jul 1;132(13):e152599. doi: 10.1172/JCI152599 (PMC9246389; doi:10.1172/JCI152599)
Supplement: Supplemental data [file jci-132-152599-s045.pdf]

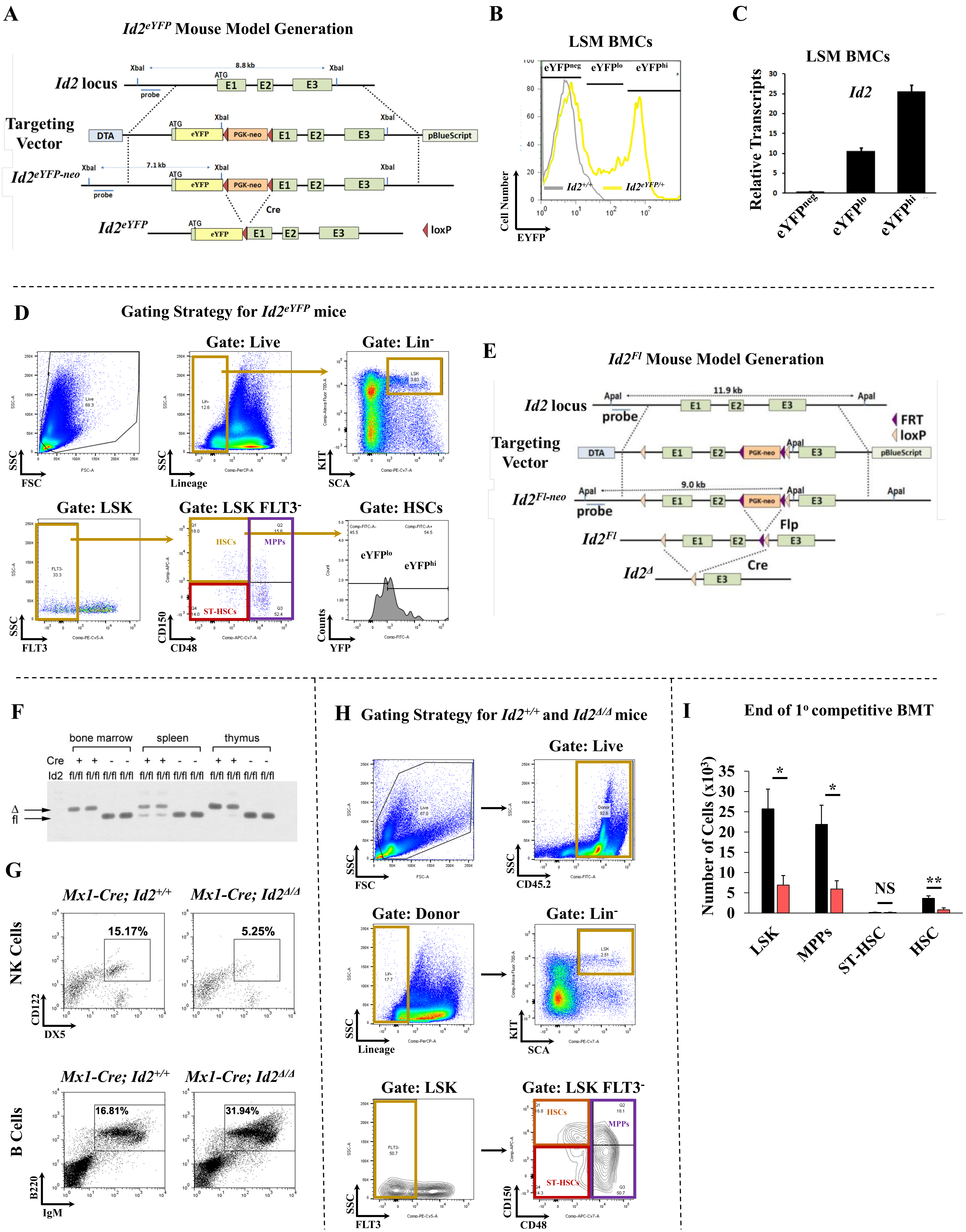

Supplemental Figure 1

Supplemental Figure 1. Generation and validation of *Id2*$^{eYFP/+}$ and *Id2*$^{Fl/+}$ mice**. A,** Schematic of the generation of *Id2*$^{eYFP/+}$ mice. **B,** ID2/eYFP expression in LSM isolated BMCs from *Id2*$^{+/+}$ and *Id2*$^{eYFP/+}$ mice. **C,** Relative *Id2* mRNA expression (upper panel) and protein expression levels (lower panel) in FACs sorted ID2^{neg}/eYFP^{neg}, ID2^{lo}/eYFP^{lo} and ID2^{hi}/eYFP^{hi} LSM BMCs. **D,** Gating strategy for ID2/eYFP expression in HSCs from *Id2*$^{eYFP/+}$ mice. **E,** Schematic of the generation of *Id2*$^{Fl/Fl}$ mice. **F,** PCR expression of floxed and deleted Id2 alleles in *Id2*$^{\Delta/\Delta}$ or *Id2*$^{+/+}$ spleen, thymus, and bone marrow following *pIpC* treatment of *Mx1-Cre; Id2*$^{Fl/Fl}$ mice. **G,** Percentages of NK cells (upper panels) and B cells (lower panels) in *Id2*$^{+/+}$ and *Id2*$^{\Delta/\Delta}$ BM. **H,** Gating strategy for HSPCs in *Id2*$^{+/+}$ and *Id2*$^{\Delta/\Delta}$ chimeric mice. **I,** Total number of HSPCs from primary BMT recipient mice.

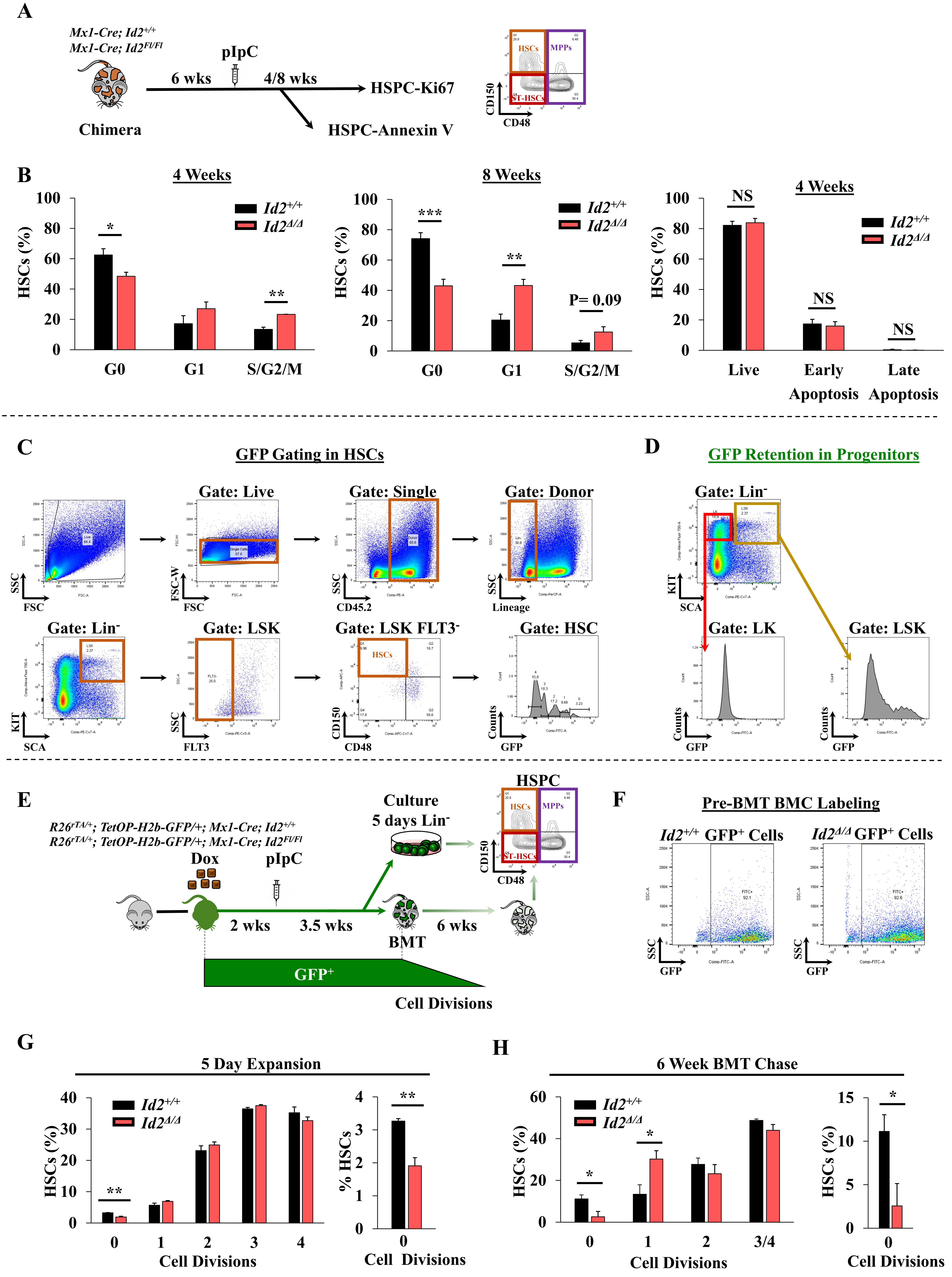

Supplemental Figure 2

Supplemental Figure 2. Cell proliferation is increased in *Id2*$^{\Delta/\Delta}$ HSCs *in vitro*. **A,** Procedure for measuring HSC cycling and apoptosis in chimeric mice. **B,** Cell cycle analysis (Ki67) of HSPCs in chimeric mice 4 (left panel) and 8 (middle panel) weeks after *Id2* deletion. Flow cytometry quantification of Annexin V stained donor HSCs in chimeric mice four weeks after *Id2* deletion (right panel). **C,** Gating strategy for GFP expression in HSPCs from *R26*$^{rtTA/+}$*;TetOP-H2B-GFP*;*Mx1-Cre;Id2*$^{Fl/Fl}$ and *R26*$^{rtTA/+}$*;TetOP-H2B-GFP*;*Mx1-Cre Id2*$^{+/+}$ mice 4 weeks after *Id2* deletion. **D,** Retention of GFP expression in LK and LSK cells after a 4-week chase. **E,** Procedure to measure the GFP retention of *R26*$^{rtTA/+}$*;Col1a-H2B-GFP*;M*x1Cre;Id2*$^{Fl/Fl}$ and *R26*$^{rtTA/+}$*;Col1a-H2B-GFP*;M*x1-Cre Id2*$^{+/+}$ labeled BMCs *in vitro* and *in vivo*. **F,** Expression of GFP in BMCs prior to *in vitro* culture and BMT. **G,** GFP retention in HSCs after a 5-day expansion culture. **H,** GFP retention in HSCs 6 weeks after chase-BMT.

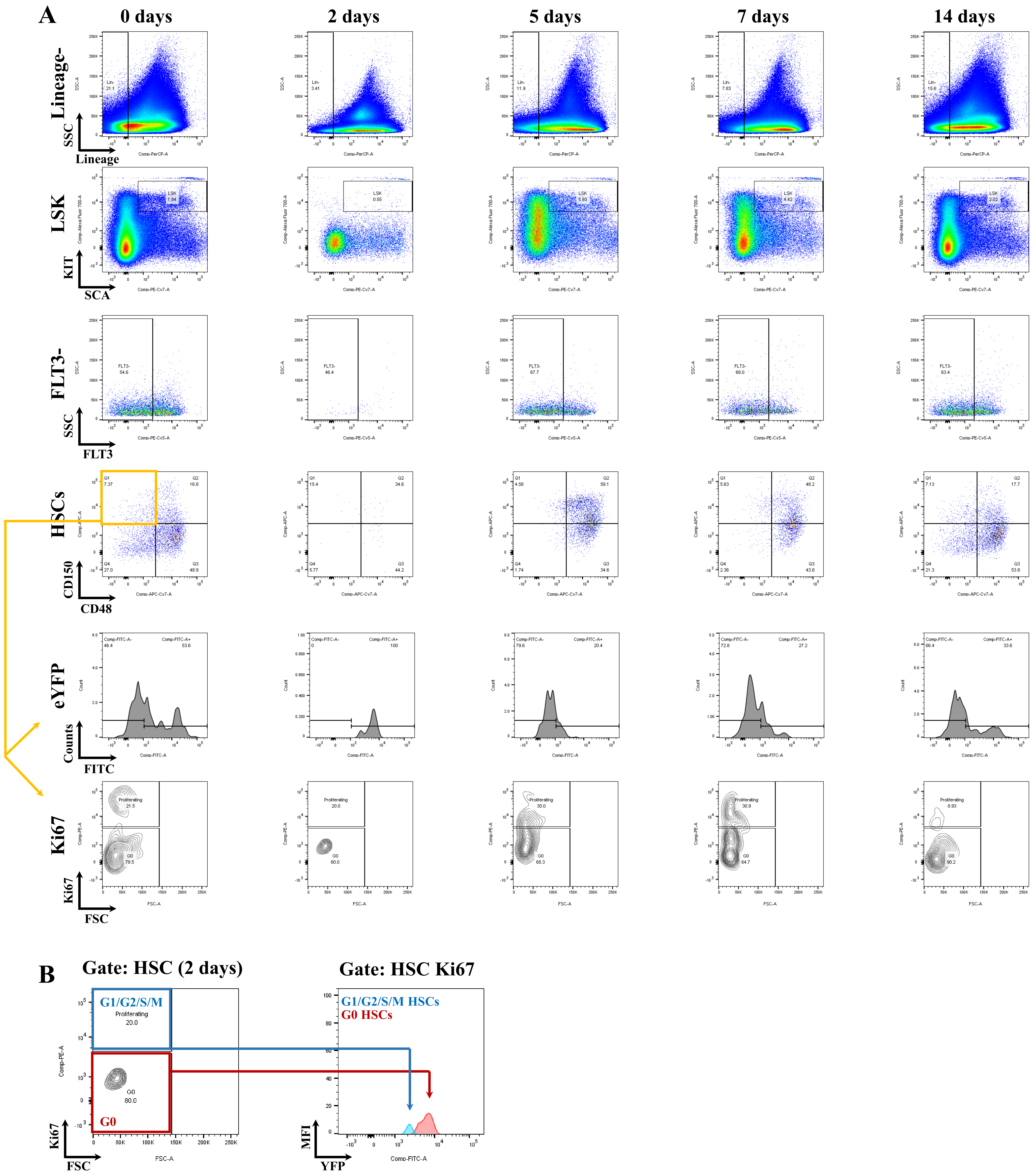

Supplemental Figure 3

Supplemental Figure 3. ID2/eYFP expression is reduced in cycling HSCs**. A,** Immunophenotype analysis of HSPCs in $Id2^{YFP/+}$ mice 2, 5, 7, and 14 days after 5-FU administration (150 mg/kg). **B,** ID2/eYFP levels in quiescent ($Ki67^{-}$) or proliferating ($Ki67^{+}$) HSCs.

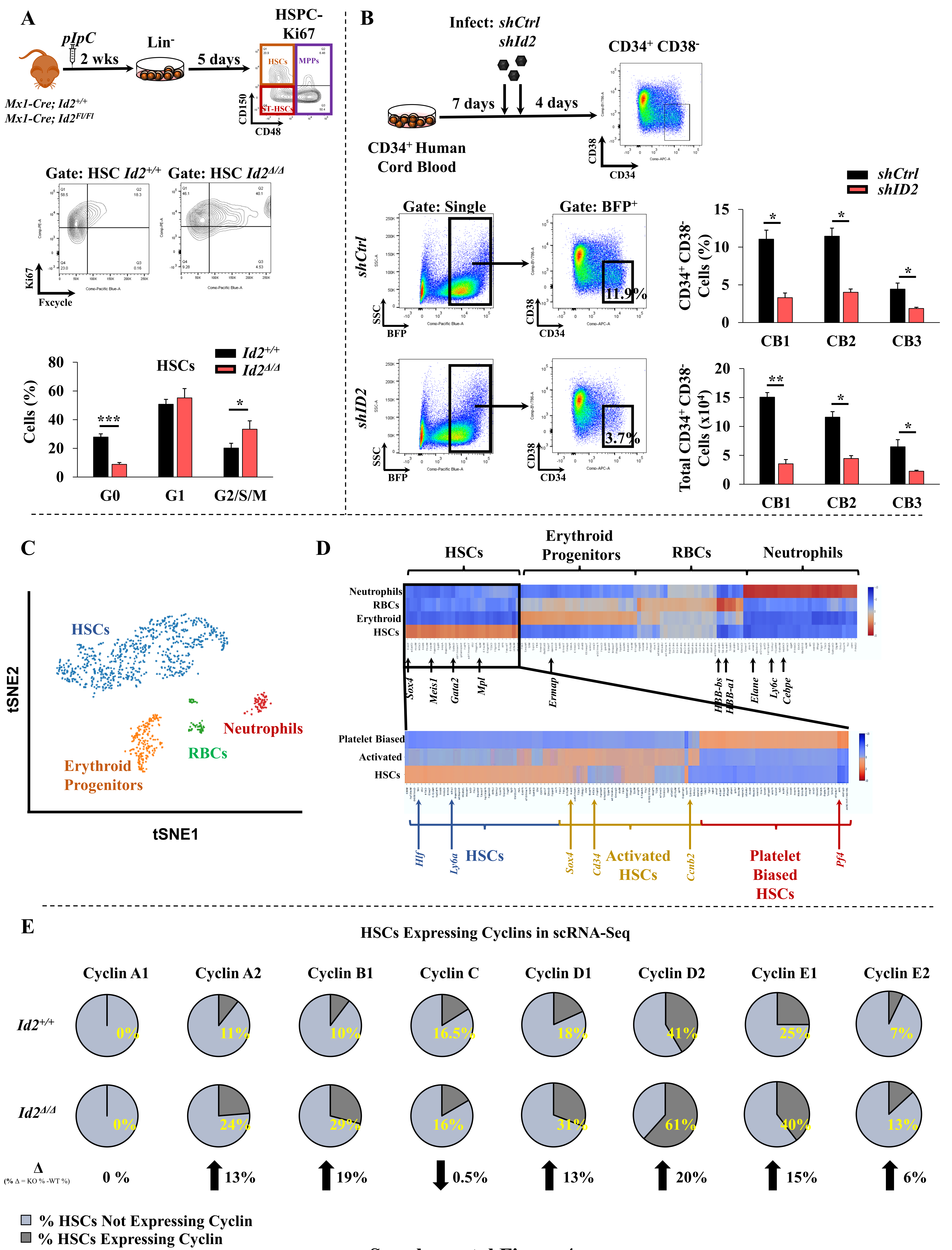

Supplemental Figure 4

Supplemental Figure 4. Summary of single-cell RNA-Seq data of *Id2*$^{+/+}$and *Id2*$^{\Delta/\Delta}$ HSCs**. A,** Cell cycle analysis of *Id2*$^{+/+}$and *Id2*$^{\Delta/\Delta}$ Lin- cells cultured in expansion medium for five days. Flow cytometry contour plots of Ki67 stained HSCs (middle panel) and quantification of *Id2*$^{+/+}$ *and Id2*$^{\Delta/\Delta}$ HSC cell cycle (lower panel). **B,** Procedure to measure CD34^{+}CD38^{-} HSPCs after KD of *ID2* expression in CD34^{+} enriched human cord blood (CB) using lentiviral shCtrl and shID2 RNA vectors (upper panel). Flow cytometry plots of shCtrl and shID2 transduced cells (BFP^{+}) and subsequent gate for CD34^{+}CD38^{-} cells in culture, and quantification of frequency (middle right panel) and total CD34^{+}CD38^{-} cells (lower right panel) from three CB samples. **C,** Cell clustering analysis from scRNA-Seq data. **D,** Heatmap of DEGs comparing HSCs, erythroid progenitors, RBCs, and neutrophils (left panel) and within HSC subpopulations (right panel). **E,** Comparison of cyclin expression in *Id2*$^{+/+}$and *Id2*$^{\Delta/\Delta}$ HSCs.

A

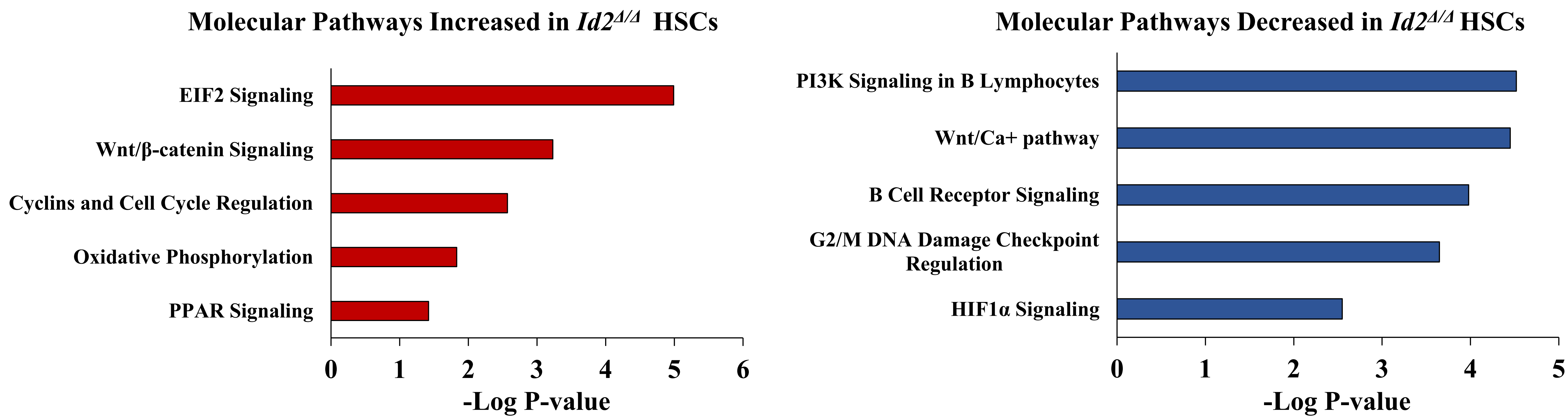

B

	Id2$^{\Delta/\Delta}$ HSC Phenotype in Chimeric KO Mice		*Id2*$^{\Delta/\Delta}$ HSC Phenotype in *Mx1-Cre;Id2*$^{Fl/Fl}$ Mice	
Assay	4 Weeks	8 Weeks	2 weeks	12 Weeks
TMRM	Increased *	Increased **	Increased ***	Increased *
ROS	Trend Increase	Increased ***	Increased *	Increased *
γH2ax	No change	Increased **	No change	Increased *

C

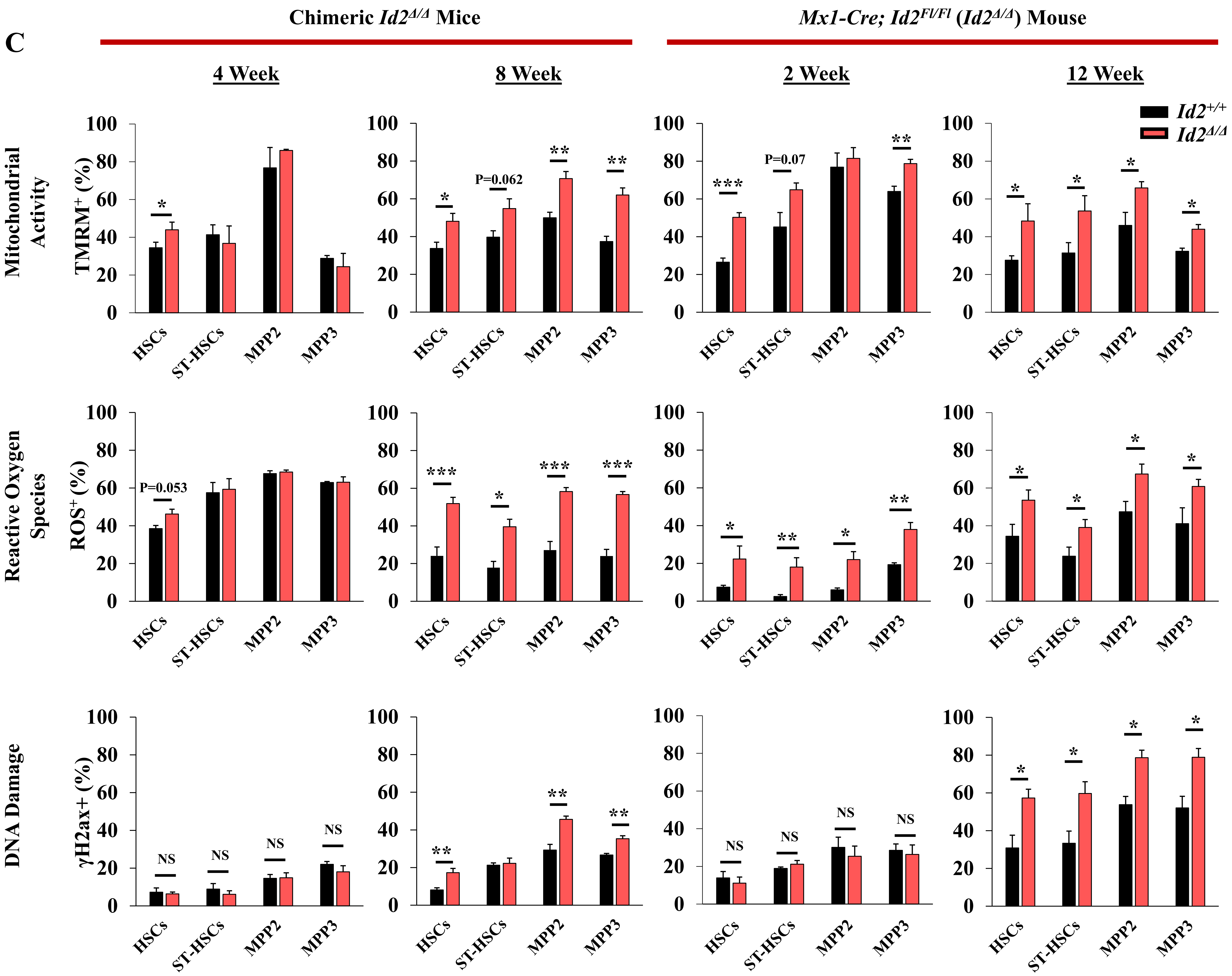

Supplemental Figure 5

Supplemental Figure 5. *Id2*$^{\Delta/\Delta}$ HSCs show increased levels of ROS, Mitochondrial Activity, and DNA damage. **A,** Ingenuity Pathway Aanalysis of *Id2*$^{+/+}$and *Id2*$^{\Delta/\Delta}$ HSCs. Summary of molecular pathways increased (left panel) and decreased (right panel) in *Mx1-Cre; Id2*$^{Fl/Fl}$ HSCs two weeks after *Id2* deletion. **B,** Summary of TMRM and ROS levels, and γH2AX phosphorylation in *Id2*$^{+/+}$*and Id2*$^{\Delta/\Delta}$ HSCs from chimeric mice 4 and 8 weeks after *Id2* deletion, and in *Mx1-Cre; Id2*$^{Fl/Fl}$ mice 2 and 12 weeks after *Id2* deletion. **C**, TMRM staining (mitochondrial activity), ROS levels and γH2AX phosphorylation of HSPCs in chimeric and *Mx1-Cre; Id2*$^{Fl/Fl}$ *Id2*$^{+/+}$and *Id2*$^{\Delta/\Delta}$ mice.

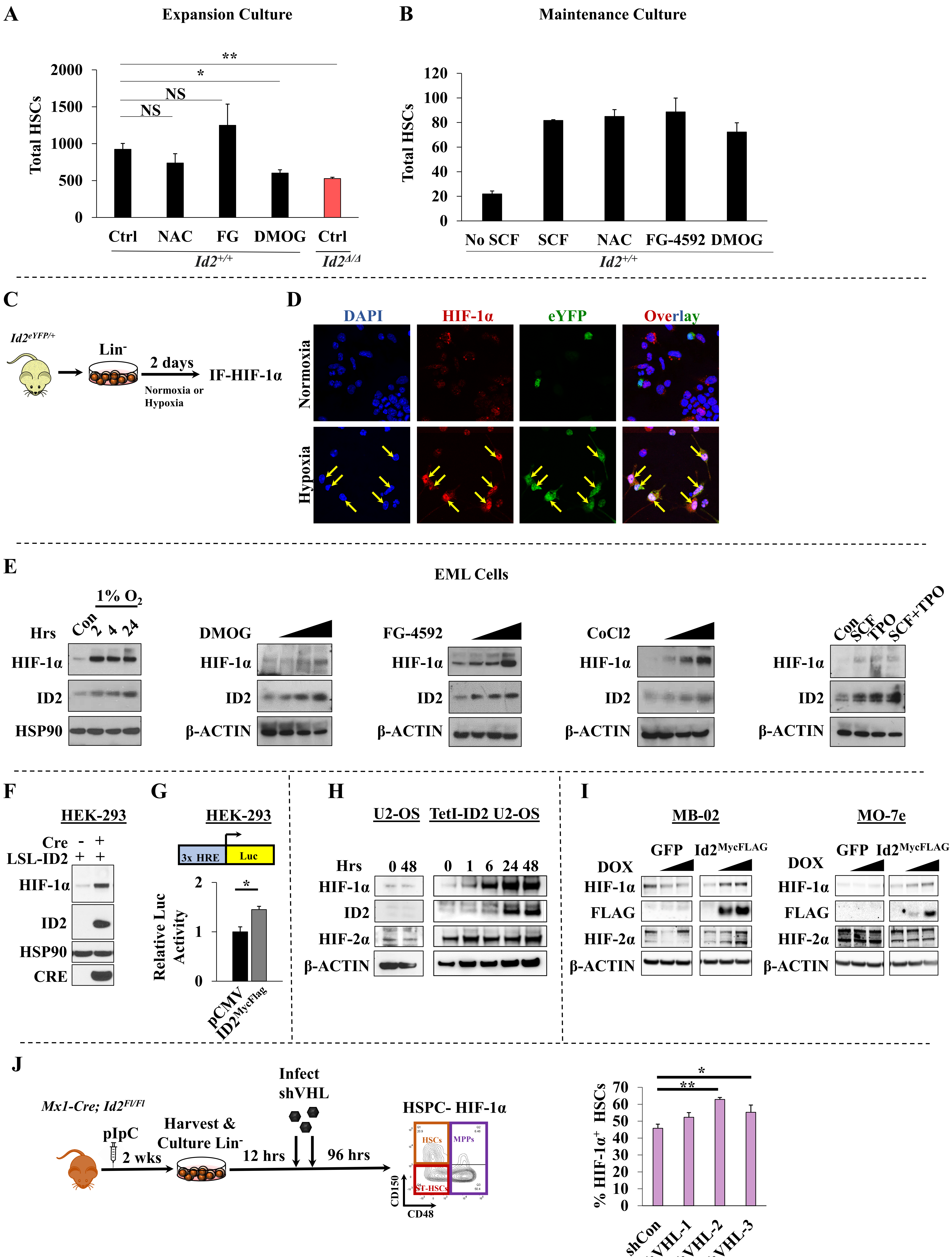

Supplemental Figure 6

Supplemental Figure 6. ID2 expression promotes HIF-1α expression and HIF-1α stabilization. **A,** Total HSCs following indicated treatments of *Id2*$^{+/+}$ HSCs in stem cell expansion assays as described in Figure 6A. **B,** Total HSCs following the indicated treatments of *Id2*$^{+/+}$ HSCs in maintenance cultures as described in Figure 7A. **C,** Schematic of *Id2*$^{eYFP/+}$ Lin^{-} cells cultured in normoxia or hypoxia. **D,** Immunofluorescent analysis of eYFP expression and HIF-1α in Lin^{-} cells. Arrows indicate same cells in multiple images. **E,** Western blot analysis of EML Cells when subjected to hypoxia for the indicated times, or PHD inhibitors DMOG (1uM, 10μM, 100 μM), $CoCL_2$ (2.5 μM, 25 μM, 50 μM) FG-4592 (0.1 μM, 1 μM, 10 μM) and cytokines SCF and TPO. **F,** Western blot analysis of HEK 293 cells transfected with a Cre-inducible ID2^{MycFlag} ± Cre. **G,** Luciferase assay of HEK 293 cells transfected with ID2^{MycFlag} or PCMV with a 3x HRE reporter. **H,** Western blot analysis of tet-inducible U2-OS cells over 48 hours. **I** Western blot analysis of hematopoietic cancer cell lines MB-O2 and MO-7e following a titration with doxycycline. **J,** Expression of HIF-1α in *shVHL* KD HSCs in culture. **A,J,** data are presented as the mean ± SEM. Comparisons between mean values of 2 groups were evaluated using an unpaired Students *t* test, and a two-way ANOVA with Dunnet's correction was used for multiple means testing in A, and a one-way ANOVA with Dunnet's correction was used for multiple means testing in J. $*P \leq 0.05$, and $**P \leq 0.01$, $***P \leq 0.001$. NS, not significant.

Supplemental Materials and Methods

Cell Lines

EML cells were maintained in Iscove's modified eagle medium 20% horse serum (Gem Cell, 100-508), 1 % penicillin/streptomycin (Gibco, 15140122), and BHK conditioned medium containing SCF at 37ºC, 5% CO_2 as described previously(1). Tet-inducible $ID2^{MycFlag}$ EML cells were generated by transduction with RRL.PPT.TII.$ID2^{MycFlag}$IRES.GFP and selected in 1 ug/ml puromycin (Sigma, P8833) and replaced every 48 hours. Clones were sorted into individual wells, expanded, and screened for GFP and $ID2^{MycFlag}$ expression using 1 ug/ml doxycycline (Sigma, D9891) for 48 hours. For HIF-1α induction assays, EML cells were cultured in 100 uM cobalt chloride (Sigma, 15862) for 16 hours or alternatively in 50 nM, 500 nM, or 5 uM FG-4592 (Cayman, 808118-40-3) or 1 uM, 10 uM, 100 uM DMOG (Sigma, D3695) for 48 hours. For hypoxia studies, EML cells were cultured in a hypoxia chamber at 1% O_2 for 2, 4, or 24 hours. HEK 293 cells were cultured in 10% Fetal bovine serum (Gibco, 15140) 1% penicillin/streptomycin in Dulbecco's modified eagle medium. Transfection studies in HEK 293 cells were performed following the guidelines outlined for Roche's Xtremegene HP DNA transfection reagent (Roche, 06366244001) in 6-well or 12-well plates. MB-O2, M-O7e, and U2-OS cells were grown as described by ATCC. Tet-inducible $ID2^{MycFlag}$ MB-O2, M-O7e, and U2-OS cells were generated by transduction with RRL.PPT.TII.$ID2^{MycFlag}$IRES.GFP and selected for in 1 ug/ml puromyocin (Sigma, P8833) and replaced every 48 hours. Clones were sorted into individual wells, expanded, and screened for GFP and $ID2^{MycFlag}$ expression using 1 ug/ml doxycycline (Sigma, D9891) for 48 hours.

Homing Assay

LSM isolated BMCs were incubated with biotin labeled lineage markers (Mac-1, Gr-1, B220, TER119, CD4, and CD8). The BMCs were depleted of lineage^{+} cells by incubating them with streptavidin beads for 2 hours (Invitrogen, Carlsbad, CA) followed by removal with a magnetic column. Lin^{-} cells were stained with 5 µM CFSE (Invitrogen) in pre-warmed PBS at 37°C for 20 minutes, washed, and transplanted into 8 Gy irradiated recipient mice (CD45.1). Recipients were euthanized 20 hrs after BMT and BMCs were analyzed by flow cytometry for CFSE+ HSPCs.

Analysis of 5-FU treated Mice

Sublethal dose of 5-FU. Chimeric mice were generated by transplanting 2.0 x 10^6 *Mx1-Cre; Id2*$^{+/+}$ or *Mx1-Cre; Id2*$^{Fl/Fl}$ BM cells into 10 Gy irradiated recipient mice. Six weeks after BMT, mice were treated with two doses of pIpC as above. Four weeks after pIpC treatment, mice were treated with two doses of 150 mg/Kg 5-flurouracil (5-FU) (NIH Pharmacy) at a one-week interval. Two weeks after the last 5-FU injection, BMCs were harvested and subjected to HSPC analysis and Ki-67 staining. To analyze HSC function, BMCs (1.0 x10^6) cells were mixed with 1.0 x 10^6 CD45.1 cells and transplanted into CD45.1 recipients. Donor reconstitution was monitored by harvesting peripheral blood 4, 8, and 12 weeks after competitive BMT. *Id2*$^{eYFP/+}$ reporter mice were subjected to one dose of 150 mg/Kg 5-FU and analyzed for Id2/eYFP and Ki-67 expression in HSPCs over the 5-FU time course. **Serial 5-FU Treatment.** Chimeric mice were generated by transplanting 2.0 x 10^6 *Mx1-Cre; Id2*$^{+/+}$ or *Mx1-Cre; Id2*$^{Fl/Fl}$ BM cells into 10 Gy irradiated recipient mice. Six weeks after BMT, mice were treated with two doses of pIpC as above. Four weeks after pIpC treatment, mice were treated with four doses of 135 mg/Kg 5-flurouracil (5-FU) (NIH pharmacy) at one-week intervals. Survival of mice was monitored

following the first 5-FU injection. Rough and lethargic mice were euthanized in accordance with the "Guide for the Care and Use of Laboratory Animals."

***Id2*$^{eYFP/+}$ Reporter Mouse Assays**

BMCs were isolated from *Id2*$^{eYFP/+}$ mice and stained with the following markers, Lineage, c-Kit, Sca-1, CD34, FcR, Mac-1, FLT3, CD150, CD48, Gr-1, CD19, IgM, CD122, NK1.1, and Dx5 and analyzed for ID2/eYFP expression by flow cytometry. For in vitro studies, BMCs were incubated with biotinylated Mac-1 (M1/70), Gr-1 (RB6-8C5), B220 (RA3-6B2), CD4 (GK1.5), CD8 (53-6.7), CD71 (R17217), IL7R and Ter119 (Ter119) and Lin^{+} IL7R^{+} cells were subsequently removed with streptavidin beads. Lin^{-} cells were then cultured in StemSpan containing msSCF (100 ng/mL) and huTPO (100 ng/mL) unless otherwise specified. All cytokines were purchased from Peprotech Inc. In vitro assays were performed using BMCs from at least three different mice for each group. BMCs were cultured for 48 hrs and HSPCs were analyzed for ID2/eYFP expression by flow cytometry. Alternatively, Lin^{-} cells were cultured in StemSpan containing msSCF, huTPO and either 1 nM, 10 nM, or 100 nM echinomycin (SML0477, Sigma). HSPC analysis was performed 24 and 48 hours after culturing. Similarly, Lin^{-} cultures were cultured in StemSpan containing msSCF, huTPO and either 2 nM, or 20 nM KC-7f2 (Selleck Chemicals, S7946).

Plasmid Constructs

ID2^{MycFlag} was received from Origene (MR200792) and cloned into RRL.PPT.TII.IRES.GFP (kindly provided by Dr. Nico Lachmann, Hannover Medical School) by amplifying *Id2* with forward primer: TAAGTCGACCGAGGAGATCTGCCGCCG, reverse primer: TAAGTCGACTTAAACCTTATCGTCGTC followed by SALI restriction digestion and standard cloning procedures. For LV.pCHD-ID2^{MycFlag}-IRES-GFP, ID2^{MycFlag} was amplified

with forward primer: TAATATTCTAGACGAGGAGATCTGCCGCCGCGATCGCCATGA and reverse primer: TAATATGGATCCCGGGCCCGCGGTACCGTCGACTTA followed by digestion with XBAI and BAMHI then by standard cloning procedures. pcDNA3.1 HIF-1α^{Myc} (P402A/P577S/N813A) was received from addgene (Plasmid #44028). For LV.pCHD-HIF-1α^{Myc}-IRES-GFP, HIF-1α^{Myc} (P402A/P577S/N813A) was amplified using the forward primer: TAAGCTAGCTCACTATAGGGAGACCCAAGC, reverse primer: TAAGGATCCTGCCCGGATCCTCACAGAT followed by digestion with BAMHI and NHEI digestion and standard cloning procedures. $ID2_{promoter}$pGL4.1 was described previously(2). $ID2_{promoter}$ HRE sites containing 5' CACA-boxes were identified bioinformatically. HRE sites were mutated using standard site-directed mutagenesis (NE, E0554S) with the site 1 forward primer: CTGTTCCGCTGTGGCGATTATGTGACAGAAAC, site 1 reverse primer: GTTTCTGTCACATAATCGCCACAGCGGAACAG and site 2 forward primer: GGCACATGGCTGTCGATTGGAGGTCAGAGACC, site 2 reverse primer: GGTCTCTGACCTCCAATCGACAGCCATGTGCC. The double mutant was created by subsequent mutagenesis of HRE site 2 in the $ID2_{promoter}$pGL4.1 HRE site 1 mutant plasmid. HRE-luciferase (HRE-Luc) expression plasmid was received from addgene (Plasmid #26731). pCAG-Cre was received from addgene (Plasmid #13775).

Luciferase Reporter Gene Assays

HEK 293 cells were transfected with pGL 4.1, 1.8 kb $Id2_{pr}$pGL4.1, or 1.8 kb $Id2_{pr}$pGL4.1 HRE mutants with pcDNA3.1, HIF-1α (P402A/P577S/N813A) (HIF-1α^{3M} – mutations that render HIF-1α resistent to VHL-mediated proteosomal degradation), and a renilla luciferase construct (pRL-pGK) described previously. Cells were cultured as described above for 48 hours. Lysates were harvested in passive lysis buffer (Promega) and analyzed on a plate reader. Firefly

luminescence was normalized internally to renilla luminescence (Fluc/Rluc ratio) then luciferase activity was compared to plasmid empty vector pcDNA3.1. Data was expressed as fold activation with HIF-1α^{3M} compared to empty vector.

Mitochondrial Activity and ROS Assays

LSM BMCs were isolated from *Mx1-Cre; Id2*$^{+/+}$ or *Mx1-Cre; Id2*$^{Fl/Fl}$ mice or respective chimeric mice following pIpC treatment. BMCs (2.5x10^{6}) were resuspended in 500 nM CellRox Deep Red (Thermo, C10422) or 100 nM TMRM (Thermo, T668) in Stemspan at 37ºC for 30 minutes, as per the manufacturer's guidelines. Cells were washed in calcium magnesium free saline and stained for HSPC markers as described in the Materials and Methods and immediately acquired on the LSR II SORP.

Hematopoietic Stem and Progenitor Expansion Assays

For in vitro studies, LSM BMCs were incubated with biotinylated Mac-1 (M1/70), Gr-1 (RB6-8C5), B220 (RA3-6B2), CD4 (GK1.5), CD8 (53-6.7), CD71 (R17217), IL7R and Ter119 (Ter119) and Lin^{+} IL7R^{+} cells were subsequently removed with streptavidin beads. Lin^{-} cells were then cultured in StemSpan containing msSCF (100 ng/mL), huTPO (100 ng/mL), huIGF2 (20 ng/mL), msFGF1 (10 ng/mL), and msAngiopoietin 2 (50 ng/mL). Cells were cultured for a period of 5 days and analyzed for HSPC cell cycling using Ki67 and BrdU, and HIF-1α expression by flow cytometry.

Lentiviral Mediated Knockdown of *Vhl*

In vitro expansion cultures were set up as described above. Mouse VHL shRNA target sequences were purchased from Sigma-Aldrich (SHCLNG-NM_009507- TRCN0000009734-TRCN0000009738). Infectious Lentivirus was generated by transfecting the shRNA constructs and packaging plasmids (PMD2G and pCMV8.74) into 293T/17 cells using XtremeGene HP

(Roche, 6366244001). Virus containing supernatants were collected 48 hours post transfection. To calculate viral titers, NIH3T3 cells were infected with serial dilutions of viral supernatants. For knockdown, Lin- cells were transduced with shRNA lentivirus by spinoculation. Briefly, Lin- cells were isolated from *Mx1-Cre; Id2*$^{Fl/Fl}$ mice 2 weeks after administration of pIpC using immune-magnetic bead separation. Lin- cells were cultured in Stemspan medium containing msSCF (100 ng/mL), huTPO (100 ng/mL), msFGF1 (10 ng/mL), huIGF2 (20 ng/mL), and Angiopoietin 2 (50 ng/mL) at 5×10^5 cells/0.5mL for 12 hours. Twelve hours after culture, the cells were subjected to first round of shRNA mediated lentiviral transduction, where lineage depleted cells were spun at 2000 x g for 90 minutes at 37°C. The cells were then washed and reseeded with fresh complete medium. After 24 hours, a second round of transduction was performed as described above, and the cells were cultured in puromycin (5 μg/mL). Ninety-six hours after the second lentiviral mediated transduction, the Lin$^-$ cells were harvested for qRT-PCR or for HSPC analysis and HIF-1α expression by flow cytometry.

Lentiviral Mediated Expression of ID2^{MycFlag} and HIF-1α^{3M}

In vitro expansion cultures were set up as described above. Cloning of LV.pCHD-HIF-1α^{3M}-IRES-GFP and LV.pCHD-ID2^{MycFlag}-IRES-GFP is described above. Lentiviral particles were generated by transfecting the LV.pCHD-IRES-GFP, LV.pCHD-HIF-1α^{3M}-IRES-GFP and LV.pCHD-ID2^{MycFlag}-IRES-GFP constructs into 293 cells as described above. Lin- cells isolated from *Mx1-Cre; Id2*$^{+/+}$ or *Mx1-Cre; Id2*$^{Fl/Fl}$ mice 2 weeks after the administration were transduced with lentivirus by spinoculation as described above. Lin- cells (5×10^5 cells/0.5mL) were cultured in Stemspan and growth factors as described above. The Lin$^-$ cells were immediately subjected to the first round of lentiviral transduction following their harvest, where cells were spun at 2000 x g for 90 minutes at 37°C. The cells were then washed and reseeded with fresh

complete medium. After 24 hours, a second round of transduction was performed as described above. Twelve hours after the second lentiviral transduction the cells were harvested and subjected to FACS. $GFP^{+/}$ LSK/ $FLT3^{-}$/$CD150^{+}$/$CD48^{-}$ HSCs were FACS sorted from LV.pCHD-IRES-GFP and LV.pCHD-$ID2^{MycFlag}$-IRES-GFP infected Lin- cells, and 20 HSCs were mixed with 50,000 CD45.1 LSM support cells and transplanted into γ-irradiated CD45.1 mice. Peripheral blood was analyzed every four weeks and BM was harvested after sixteen weeks and subjected to HSPC analysis.

Western Blot Analysis

Protein lysates were harvested using RIPA buffer (Pierce, 89901) containing protease inhibitor cocktail (Roche) and phosphatase inhibitors (Active Motif). Lysates were vortexed, pelleted and supernatant was taken following centrifugation. For BM lysates, LSM BMCs were isolated and prepared as above. PVDF membranes were incubated at RT with antibodies: C-myc (1:1000 Santa Cruz, 9E10), ID2 (1:1000 Santa Cruz, C-20), ID2 (1:1000 Abcam, ab85990), α/β HSP90 (1:5000 Santa Cruz, SC-7947), HIF-1α (1:1000 Abcam, ab179484), VHL (1:1000 Santa Cruz, SC-17780), VHL (1:1000 GeneTex, GTX101087), Flag (1:1000 Sigma, F3165), HIF-2α (1:1000 abcam, ab8365), and β-actin (1:5000 Sigma, A5541) for 1 hr. Membranes were washed and incubated with HRP-conjugated anti-mouse and anti-rabbit antibodies (1:5000 Vector, P1-1000). ECL (Thermo, 34087) was used for peroxidase reactions.

Co-Immunoprecipitation Assays

Protein lysates were harvested in low salt lysis/binding buffer containing protease inhibitor cocktail (Roche) and phosphatase inhibitors (Active Motif). Samples were lysed via light sonication. Samples were subjected to immunoprecipitation using anti-IgG Rabbit (Cell signaling, 2729), anti-IgG Mouse (Santa Cruz, SC-2025), anti-ID2 (Abcam, ab85990), anti-VHL

(GeneTex, GTX101087) and anti-Flag (Sigma, F3165) bound to streptavidin bound beads associated with biotinylated anti-mouse IgG (Vector, BA-2001) and biotinylated anti-Rabbit IgG (Vector, BA-1000). Samples were incubated overnight, washed 3x, eluted using SDS buffer, and analyzed by estern blot analysis.

Gene Expression Analysis

RNA was extracted from LSK/ $FLT3^-$/$CD150^+$/$CD48^-$ sorted cells or EML cells using either the RNeasy micro Kit (Qiagen, 74004) or the RNeasy Mini Kit (Qiagen, 74104). cDNA synthesis was performed using either 50 ng or 1 ug RNA with the iscript cDNA synthesis kit (Bio-Rad, 1708890). Quantitative RT-PCR was performed using the Step-one Plus system with SyBr green (Roche, 04913850001). Amplicons were sequence verified. Genes were normalized to either 18S rRNA or β-actin. Primer sequences can be found in Supplemental Table 1. Three biological replicates were performed in each assay with two technical replicates for each sample. Data was analyzed using the standard ΔΔCT method to determine expression changes.

RNA-Seq Analysis

RNA was purified from HSCs isolated from *Mx1-Cre; Id2*$^{+/+}$ or *Mx1-Cre; Id2*$^{Fl/Fl}$ mice by flow cytometry as described above, and from HSCs isolated from mice 2 weeks after pIpC treatment. The RNeasy™ Micro Kit (Qiagen, 74004) was used to generate high quality RNA. The sample was quantified and sequenced on the Illumina HiSeq 2500 sequencer. The HiSeq Real Time Analysis software (RTA 1.18) was used for processing image files, the Illumina CASAVA_v1.8.4 was used for demultiplex and converting binary base calls and qualities to fastq format. The sequencing reads were trimmed for adapters and low-quality bases using Trimmomatic (version 0.30). The trimmed reads were aligned to mouse mm9 reference genome (NCBIM37 /UCSC mm9) and Ensembl annotation version 67 using TopHat_v2.0.8 software.

Quantification was carried out with RSEM using the transcriptome bam file created by STAR. Differentially expressed genes (DEGs) were analyzed with Ingenuity Pathway Analysis (IPA) software (Ingenuity Systems, Inc., Redwood City, CA, USA) and Gene Set Enrichment Analysis (GSEA, Broad Institute, Cambridge, MA, USA). For IPA, pathways above a log P-value of 1.33 and a Z-score above 1 were considered statistically significant. For GSEA pathways, Normalized enrichment scores ≥ 1.3 and a FWER p-value below 0.05 were considered significant.

Single Cell RNA-Seq Analysis

Approximately 1,000 HSCs (Lin$^-$ Sca-1$^+$, Kit$^+$, FLT3$^-$, CD150$^+$, CD48$^-$) were sorted from *Mx1-Cre; Id2*$^{+/+}$ or *Mx1-Cre; Id2*$^{Fl/Fl}$ mice and captured using the 10X Genomics Chromium, as previously described (http://software.10xgenomics.com/single-cell). The number of cells captured ranges from 384 to 686 and mean reads per cell ranged from 218,801 to 550,748. Cells with extremely low number of UMI counts were filtered out. Median genes found per cell ranges from 2,687 to 3,274 and the total number of genes detected ranges from 14,556 to 15,256. All metrics are within the expected range for 10x Chromium single cell libraries. The single-cell barcoded cDNA libraries were sequenced on NextSeq500 (Illumina) for two runs. The sequencing run was setup as a 26 cycles + 57 cycles in a non-symmetric run. The 10X Genomics Cellranger toolkit (v2.2.0) was used to perform de-multiplexing, allowing 1 mismatch in the barcodes, and alignment to the mm10 transcriptome and gene-barcode matrices. Pre-processing, removal of highly variable genes and contaminating immune cells, dimensionality reduction, and clustering analyses procedures were applied to each data set and merged data sets using the Seurat R package (Satija 2015, Nat. Biotech.) https://satijalab.org/seurat/v3.0/pbmc3k_tutorial.html). In brief, cells that had feature counts

<200, >2500, or >5% mitochondrial counts were removed. Mean reads/cell, median genes/cell, and number of removed and analyzed cells for each data set are reported in Figure S6C. Data was then normalized, multiplied by a scale factor of 10,000, and log-transformed. Dimensionality reduction was performed via principal component analysis followed by clustering t-SNE visualization. Differentially expressed genes, which represented at least 25% of each cluster ($p<0.05$), were initially visualized using the *Heatmap* function then further analyzed using Loupe Cell Browser for gene set enrichment and pathway analysis.

Immunofluorescence Analysis of Sorted HSCs and Cultured BMCs

Bone marrow cells from *Mx1-Cre; Id2*$^{+/+}$ or *Mx1-Cre; Id2*$^{Fl/Fl}$ mice were harvested two weeks post pIpC treatment and subjected to a LSM density gradient. HSCs were stained with cell surface markers and LSK/FLT3^{-}/CD150^{+}/CD48^{-} cells were isolated using FACS. HSCs (1 x 10^{3}) were plated on pre-treated rectronectin (20 ug/ml) coated chamber slides (Thermo Fischer Scientific). HSCs were grown in Stemspan medium (Stemcell Technology, 09650) supplemented with murine stem cell factor (msSCF) (Peprotech, 250-03) and thrombopoietin (TPO) (Peprotech, 300-18). For HIF-1α staining, cells were allowed to adhere to the coverslip for 20 minutes at 37°C 20 % O_2, at which point cells were either subjected to normoxia (20% O_2) or Hypoxia (1% O_2) for two hours. For Numb staining, cells were grown for 16 hours at 37°C 20 % O_2. HSCs were then treated with 10 nM Nocodazole for 24 hours. All assays were fixed with 1% paraformaldehyde for 15-30 minutes at RT, washed with PBS and permeabilized in 0.2% Triton-X 100 for 10 minutes. Cells were blocked in either 5% BSA or M.O.M. blocking reagent (Vector SP-2001) for 1 hour at RT. HSCs were stained for HIF-1α or Numb (Abcam, ab14140) using 1:100 dilutions for 1 hour at RT. Cells were washed 3x in PBS and stained with anti-rabbit FITC with a 1:200 dilution for 30 minutes at RT. Cells were mounted on slides with

Vectashield (Vector H-1500) containing DAPI. Cells were imaged using the LSM 780 imaging system equipped with Spinning Disk Confocal CSU-W1 and Zyla4.2 sCMOS camera using the 63x oil immersion lens. Images were processed in Zeiss Black software and analysis was performed using ImageJ.

ATP Assay – Hematopoietic Stem Cells

For intracellular ATP levels, LT-HSCs (LSK FLT3$^-$ CD150$^+$ CD48$^-$) were isolated from *Mx1-Cre; Id2*$^{+/+}$ or *Mx1-Cre; Id2*$^{Fl/Fl}$ mice 4 weeks post *pIpC* treatment by FACS sorting as described above. HSCs (3x10^3) were analyzed for intracellular ATP using CellTiter-Glo 2.0 (Promega, G9241) following the manufacturer's guidelines.

H2B-GFP HSC Division Assays

Chimeric *R26*$^{rtTA/+}$*; TetOP-H2b-GFP; Mx1-Cre; Id2*$^{Fl/Fl}$ and *R26*$^{rtTA/+}$*; TetOP-H2b-GFP; Mx1-Cre; Id2*$^{+/+}$ mice were placed on doxycycline chow 2 weeks post BMT and kept on the doxycycline chow for a period of 6 weeks. Mice were injected with pIpC one week prior to removal to doxycycline. Four and 8 weeks after doxycycline removal, BMCs were harvested from mice for HSPC analysis and retention of GFP signal. Alternatively, *R26*$^{rtTA/+}$*; TetOP-H2b-GFP; Mx1-Cre; Id2*$^{Fl/Fl}$ and *R26*$^{rtTA/+}$*; TetOP-H2b-GFP; Mx1-Cre; Id2*$^{+/+}$ mice were subjected to a doxycycline diet for 6 weeks. Mice were injected with pIpC after 4 weeks on doxycycline, and then BMCs were harvested 2 weeks after pIpC injections for BMT to generate chimeric mice, or purify Lin- for in vitro expansion cultures. BMCs from chimeric mice were harvested 6 weeks after BMT and LSM BMCS were analyzed for HSPCs and retention of GFP signal.

Single-Cell Division Kinetics Assays

BMCs were harvested from *Mx1-Cre; Id2*$^{+/+}$ or *Mx1-Cre; Id2*$^{Fl/Fl}$ 2 weeks after treatment with pIpC. Single LSK FLT3$^-$CD150$^+$CD48$^-$ HSCs were sorted directly into four *Id2*$^{+/+}$and *Id2*$^{\Delta/\Delta}$

Terasaki plates (72 wells) (Greiner Bio-one) in Stemspan containing either msSCF (100 ng/mL) and huTPO (100 ng/mL) or msSCF (100 ng/mL), huTPO (100 ng/mL), and msIL-3 (30 ng/mL). Cells were counted every 12 hours up to 60 hours and analyzed again at 10 days. Cells were supplemented with fresh medium every three days. Total cells and cell size at 10 days were analyzed using ImageJ software.

Statistics.

Statistical significance was determined using unpaired Student *t*-tests using Welch's correction when applicable. Kaplan-Meier survival studies were analyzed using the log-rank test. *In vivo* studies were performed using N=5 mice for each group and were repeated two or more times using an additional N=3-5 mice. *In vitro* studies performed used a minimum N=3 and were repeated 2 or more times. $P \leq 0.05$ was considered statically significant. Error bars portray the standard error of the mean data. *$P \leq 0.05$, **$P \leq 0.01$, ***$P \leq 0.001$.

Study approval.

Experiments involving the use of mice were approved by the NCI at Frederick Animal Care and Use Committee in accordance with the eighth edition "Guide for the Care and Use of Laboratory Animals."

REFERENCES

1. Ji M, Li H, Suh HC, Klarmann KD, Yokota Y, and Keller JR. Id2 intrinsically regulates lymphoid and erythroid development via interaction with different target proteins. *Blood.* 2008;112(4):1068-77.

2. Li H, Ji M, Klarmann KD, and Keller JR. Repression of Id2 expression by Gfi-1 is required for B-cell and myeloid development. *Blood.* 2010;116(7):1060-9.
